# Identification of Priority Nutrients in the US: Targeting Malnutrition to Address Diet-Related Disease Across the Lifespan

**DOI:** 10.3390/nu17121957

**Published:** 2025-06-09

**Authors:** Carlene S. Starck, Tim Cassettari, Emma Beckett, Emily Duve, Flavia Fayet-Moore

**Affiliations:** 1 FOODiQ Global, Sydney, NSW 2000, Australia; tim@foodiq.global (T.C.); emma@foodiq.global (E.B.); emily@foodiq.global (E.D.); flavia@foodiq.global (F.F.-M.); 2 School of Health Sciences, The University of New South Wales, Sydney, NSW 2033, Australia; 3 School of Environmental and Life Sciences, The University of Newcastle, Callaghan, NSW 2308, Australia

**Keywords:** malnutrition, diet-related disease prevention, chronic disease, priority nutrients, inadequate nutrient intake, increased nutrient requirements

## Abstract

**Background/Objectives:** Poor diet is a leading modifiable cause of chronic disease in the US. In addition to targeting nutrients of concern (saturated fat, added sugars, and sodium), nutrients with both inadequate intakes and associations with major health outcomes require identification. We aimed to identify priority nutrients to address both malnutrition and diet-related disease in the US population. **Methods:** An established method for identifying priority nutrients across multiple demographic groups was adapted for the US population. This method evaluates and scores nutrients consumed at insufficient or excessive levels, with proposed revised requirements, and shows associations with established health priorities, based on the degree of deviation from recommendations and the number of linked health priorities. Priority nutrients were defined as those scoring in the top 25%. For each priority nutrient, a comparison of intake levels against the Dietary Reference Intake (DRI) was conducted. **Results:** There were 21 of 24 nutrients with consumption below recommended levels in at least one demographic group. Certain nutrients, such as dietary fiber, vitamin D, and choline, exhibited particularly high inadequacy rates, exceeding 90% throughout different life stages. The highest priority nutrients included vitamin D, vitamin E, calcium, magnesium, and dietary fiber, with vitamin D, omega-3 fatty acids, zinc, folate, and potassium showing priority for specific demographic groups. Comparing current intake levels with those known to benefit health priorities indicated that higher intakes of vitamin D, vitamin E, and calcium could be beneficial. **Conclusions:** Ten essential nutrients play a role in the prevention of diet-related disease, yet are consumed inadequately across the US population, suggesting that the prioritization of these nutrients can help to address the burden of chronic disease. Priority nutrients should be considered in diet and nutrition policies and guidelines.

## 1. Introduction

Poor diet is a leading modifiable cause of mortality and morbidity in the United States of America (US) [1]. Poor diet is directly related to both malnutrition (inadequate, excess, or imbalanced nutrient intake) and diet-related chronic diseases, including (but not limited to) type 2 diabetes (T2D), cardiovascular disease (CVD), obesity, and some cancers [2,3]. The issue of nutrient inadequacy is significant [4] with multiple key nutrients, as well as the foods and dietary patterns that support these nutrients being under-consumed [3,5,6]. Such inadequacies are globally prevalent [4], expected to worsen over time [7], and correlate with rising rates of chronic disease [8]. While current efforts to address poor diet and epidemic levels of diet-related disease are largely focused on excessive energy, sodium, sugars, and saturated and trans fats [9,10], there is a recognized need for further action in the US, including evidence-based policy solutions [11]. Current strategies, as recommended by health professionals, nutrition scientists, and policy-makers, include implementing a “food as medicine” approach, where nutrition guidelines and dietary prescriptions are targeted towards the individual, their food choices, and priority health issues [11]. An essential step towards tackling both malnutrition and diet-related disease in the US involves identifying the nutrients that should be prioritized, considering both inadequate intakes and the association with specific health outcomes, for each key demographic group.

The prioritization of nutrients to address both malnutrition and diet-related disease has been published for Australia (AUS) and New Zealand (NZ) [12]. This research identified 22 nutrient inadequacies across demographic groups. Some of these inadequate nutrients show associations with high priority diet-related health outcomes across the lifespan; for example, dietary fiber with cancer [13,14] and cardiometabolic health [15], calcium with bone growth during childhood and bone mass maintenance with aging [16,17], and vitamin D with cognitive function [18], breast cancer risk [19], depression [20], and metabolic health [21]. Within the AUS/NZ data, nine nutrients (vitamin D, calcium, omega-3 fatty acids, magnesium, folate, dietary fiber, iron, selenium, and zinc) showed a high priority in at least one demographic group, based on the specific level of intake and health priorities of that group. Six nutrients (vitamin D, calcium, omega-3 fatty acids, magnesium, folate, dietary fiber) were classified as a priority across the entire population, indicating that a set of nutrients could benefit all individuals, regardless of sex and/or age, informing both national and demographic-specific recommendations and guidelines. The application of this methodology to the US population can uncover nutrients with high potential to affect meaningful benefits in diet-related diseases, both across the lifespan (for each demographic group) and for the overall population.

Meeting Dietary Reference Intakes (DRIs) is used as a measure of nutrient adequacy. However, even if recommended intakes are met, nutrient inadequacy may also be present in instances where there is reduced nutrient absorption, bioactivity, and/or increased excretion. In addition, increased intakes of some nutrients may provide health benefits beyond a frank deficiency. For example, the maintenance requirement for protein has been suggested to be too low, based on the re-evaluation of nitrogen balance data, new data from isotope tracer studies [22,23,24], and the influence of increased protein intake on clinical functional outcomes such as sarcopenia [24]. In AUS/NZ, suggested dietary targets (SDTs) for the prevention of chronic disease have been developed for some nutrients [25], representing increased requirements relative to reference values that may provide additional benefit. Priority nutrient recommendations need to be accompanied by knowledge of the magnitude of intake that can support not only inadequacy, but also the prevention of diet-related disease, including the possibility that increased intake targets may be necessary for some nutrients, and specific to demographic groups and/or health outcomes. The identification of priority nutrients in the American population will highlight the specific nutrients that can be targeted across demographic groups for meaningful impacts on population health, thus helping to inform research and policy.

The objective of this research is to use our previously published methodology [12], adapted to the American context, to determine the priority nutrients to address to contribute to the prevention of malnutrition and chronic disease for the US population overall, and for specific life stages, represented by demographic groups (age/sex). It was hypothesized that nutrient shortfalls would be prevalent, and that each demographic group would be characterized by a specific set of priority nutrients. We also hypothesized that a select set of key nutrients would show relevance to malnutrition and diet-related disease across the entire lifespan.

## 2. Materials and Methods

### 2.1. Conceptual Summary

**Figure 1:**
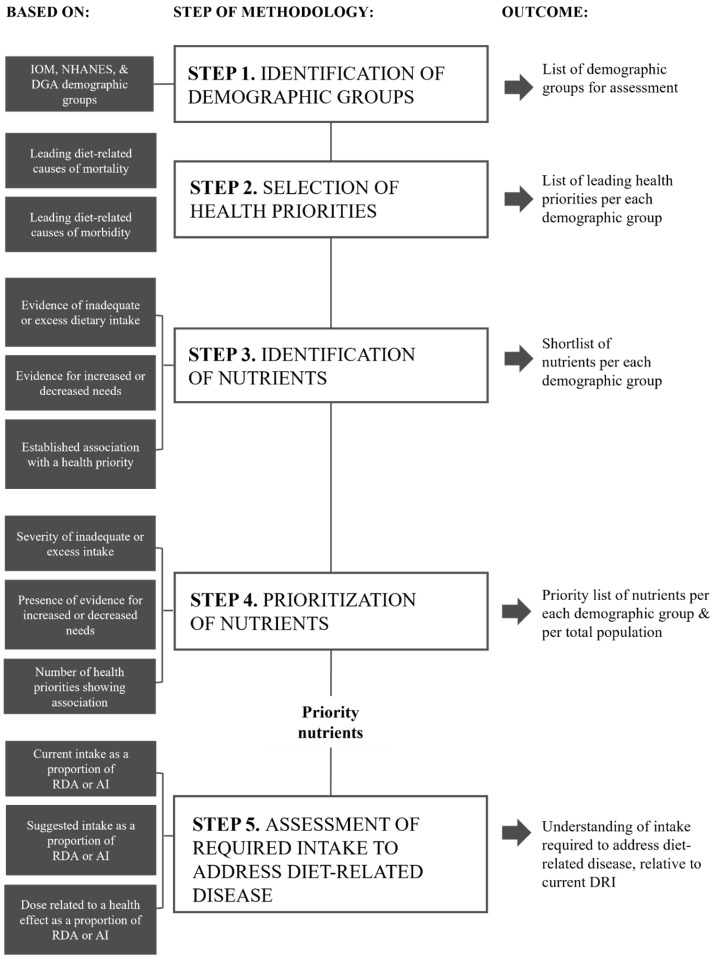
Overview of the five-step methodological approach used to identify priority nutrients for the US.

The purpose of this research was to undertake a holistic assessment of nutrients with the highest potential to concurrently address both malnutrition and diet-related disease in the US population, across the lifespan. A five-step methodological approach, based on the four-step method used to identify priority nutrients for the AUS/NZ populations, as previously described by Starck et al. [12] was reproduced (Figure 1). In addition to modification for the US context, a fifth step was added to investigate the intake of each priority nutrient identified and compare this intake to current recommendations, to determine if increased dietary targets specific to disease prevention may be of benefit. Each of the five steps is outlined in Figure 1.

### 2.2. Identification of Demographic Groups (Step 1)

Demographic groups were defined based on the life stages used by the National Academies Institute of Medicine Dietary Reference Intakes (DRIs) [26], National Health and Nutrition Examination Survey (NHANES) nutrient intake data [27,28] and the Dietary Guidelines for Americans [29]. The final selected demographic groups considered both consistency in DRIs across ages and sexes, as well as the biological needs of each life stage and their clinical relevance for nutrient requirements. Age and sex groupings were collapsed if there were no clinically relevant differences in nutrient requirements between sexes of the same age group or consecutive age groups. Age and sex groupings were split if one or more sub-populations of a group were differentiated biologically.

### 2.3. Selection of Health Priorities (Step 2)

The approach applied to the selection of health priorities for each demographic group was developed to allow for the identification of a broad scope of health outcomes (including both physical and psychological conditions), thus aligning with the holistic intent of the research. Health priorities for each demographic group were informed by national US data collected by the Centers for Disease Control and Prevention (CDC) [30,31], the health priorities laid out by the US Department of Health and Human Services [32], and an examination of the scientific literature [33,34,35]. Health priorities were selected if they were the leading causes of death, non-fatal physical morbidity, and mental or cognitive ill-health, which are known to be modifiable by diet. Health outcomes existing as a subtype of a more significant disease or set of health issues were collapsed within one umbrella health outcome. Health outcomes with the highest prevalence of mortality and/or morbidity and the highest association with diet were prioritized. The maximum number of included health priorities for any one demographic group was five. This number was selected based on balancing multiple goals: to focus on major health outcomes, maintain a manageable and concise scope of research, and allow for the specific health priorities for each demographic group to be identified. To incorporate the large number of diet-related health issues and diseases identified as a national priority for older adults, cognitive function, bone health, and sarcopenia were collapsed within one group defined as ‘independence’.

### 2.4. Identification of Nutrients (Step 3)

Nutrients included in the assessment (N = 24) were those with an Estimated Average Requirement (EAR) or Adequate Intake (AI) outlined by the Institute of Medicine DRIs [26,36,37] (Table 1).

The omega-3 fatty acids docosahexaenoic acid (DHA) and eicosapentaenoic acid (EPA) were added based on the previous AUS/NZ research [12] showing consistent associations with high-priority health outcomes. As per the previous methodology, the following exclusions were observed: energy, due to its dependence on more than one nutrient; sodium, saturated fat, and sugars, due to being established nutrients of concern in the population; water, due to the impact of additional beverages, including those containing nutrients, on hydration status. Pantothenic acid, biotin, chromium, fluoride, manganese, and molybdenum were not included due to an absence of national intake data. For each of the included nutrients per each demographic group, evidence was collected in three areas: 1. Evidence of inadequate or excess dietary intake (as mean intake and percentage of the demographic group with intake less than the EAR or AI, as applicable, or greater than the Tolerable Upper Intake Level (UL), respectively); 2. Scientific evidence for increased or decreased needs, including that addressed within the current DRIs (such as limited utilization by a demographic group) and where a revision of requirements (relative to the NRVs) has been suggested in the literature; and 3. An established association (beneficial or adverse) with one or more of the health priorities identified.

#### 2.4.1. Dietary Intake: Inadequate or Excess

Intake data were obtained from the What We Eat in America (WWEIA) database [28], the dietary component of the NHANES dataset. NHANES dietary intake data were initially collected by trained interviewers using the 5-step USDA Automated Multiple-Pass Method (AMPM) over two days of 24 h dietary recall. The Day 1 interview was conducted in person, and the Day 2 interview was conducted by telephone 3 to 10 days later, on a different day of the week from the Day 1 interview. To identify inadequate or excess nutrients, the most recent intake data available for each nutrient, including information provided within the dataset regarding the proportion of the population with intake below the EAR or AI, or above the UL, were used. For the majority of nutrients, this was the NHANES 2017 to March 2020 pre-pandemic data release [27]. For pregnant and lactating women, the most recent NHANES data are 2003–2006 for select nutrients (dietary fiber, vitamin D, calcium, phosphorus, and magnesium) [28]; this was supplemented by a targeted literature search via the PubMed database for peer-reviewed original research of nationally representative US data. More recent NHANES datasets have excluded pregnant and lactating women due to the sample being too small to allow for reliable estimates. For iodine, intake data from the Global Dietary Database were accessed [38]. These data include median intake and 5th and 95th percentile intakes for five-year age and sex groupings.

For each nutrient and each demographic group, mean intake data and the proportion of the population not meeting the EAR or AI or exceeding the UL (where appropriate) were obtained, as provided within the NHANES reports. For iodine, the percentile of intake corresponding to the EAR was approximated using the mean intake data, 95% confidence interval, and z-score tables, according to the formula: z = (x − µ)/σ, where z = z-score, x = EAR, µ = mean, and σ = standard deviation, calculated as the 95% confidence interval divided by 3.92.

#### 2.4.2. Evidence for Revised Needs: Increased or Decreased

Evidence for revised (increased or decreased) nutrient needs was defined as there being consensus in the scientific literature that the DRI (EAR, RDI, or AI) is too low or high for a demographic group, or that the demographic group may benefit from intakes above or below the specified requirement. Evidence was obtained via a targeted literature search of peer-reviewed studies using the PubMed database. Consensus in the scientific literature was defined as consistency in relevant reviews, randomized controlled trials, balance studies, or a position statement from a national or global authority providing a sound rationale for an increased or decreased requirement of a specific nutrient relative to the DRIs. For some identified nutrients, the rationale and evidence for increased needs were already accounted for by the DRIs and therefore reflected in the level of inadequacy reported by the collected dietary intake data. In these cases, the relevant nutrients were not included as having increased needs to avoid double scoring in the following step (Step 4).

#### 2.4.3. Health Priority Association: Beneficial or Adverse

A targeted literature search of the PubMed (with Medline) database was undertaken for systematic literature reviews (SLRs) of primarily randomized controlled trials (RCTs) or prospective cohort studies showing an association of a nutrient with one of the identified health priorities. The search was focused on capturing the most recent and highest-level evidence for each nutrient and health priority combination up to September 2024, rather than all evidence published to date. Evidence was limited to the demographic group or groups with that specific health priority, according to the age ranges covered by the included research. Search strategies are listed in Supplementary Table S1. Only SLRs of RCTs or prospective cohort studies were eligible for inclusion to ensure that the highest level of available evidence was captured, based on the Oxford Centre for Evidence-Based Medicine (OCEBM) framework [39]. The search and data selection include both beneficial and adverse associations. Neutral and inconclusive associations were not included. Associations for sub-groups (e.g., vegetable vs. cereal fiber and animal vs. plant protein) were not considered. For example, where a systematic review assessed associations between total fiber, as well as fiber from individual food sources (such as fruits, vegetables, nuts and seeds, and cereal foods), association data for total fiber only were used. Where multiple SLRs reported conflicting findings for the same nutrient-health priority association, the evidence was considered to be inconclusive.

### 2.5. Prioritization of Nutrients (Step 4)

Nutrient prioritization was carried out using a points-based scoring matrix as previously described [12], with adaptations (Table 2). Each nutrient in each demographic group was awarded points in each of the three areas to produce three sub-scores: dietary intake (inadequate or excess), revised needs (increased or decreased), and overall association with health (beneficial minus adverse). The three sub-scores were summed to produce a total priority score. The dietary intake sub-score was calculated to reflect the magnitude of inadequacy or excess of a demographic group relative to the EAR or AI (at least 20% or 50% not meeting the EAR or AI, as applicable, or exceeding the UL, respectively). A demographic group contained more than one age/sex group, and scoring was based on the highest level of inadequacy or excess to ensure that the most at-risk group was accounted for. When the percentage inadequacy or percentile data were not available, a mean intake less than or greater than the DRI for that group was taken to represent inadequacy or excess, respectively.

Up to one additional point was awarded for evidence of increased or decreased needs, as appropriate, and up to five additional points were awarded for established effects on one or more health priorities. The health association sub-score was the difference between the score for beneficial associations and that for adverse associations. The maximum and minimum total priority scores for any nutrient were 10 and −10, respectively. Positive scoring represented a beneficial nutrient, whereas negative scoring was taken to represent a nutrient of concern. The highest priority nutrients for a demographic group were defined as those scoring in the top 25% for that group, based on the absolute score (positive or negative). The highest priority nutrients across the entire population (all included demographic groups) were determined by summing the total scores per nutrient across all demographic groups. A maximum score of 80 was achievable for a population-level priority nutrient. Where multiple nutrients achieved the same score, preventing clear distinction according to the number of nutrients in the top quartile, the priority nutrient to be included was based on sub-score values: that with the highest level of inadequate or excess intake in the first instance, followed by the highest number of health associations, and then the presence of evidence for increased or decreased needs.

### 2.6. Assessment of Required Intake to Address Diet-Related Disease (Step 5)

The purpose of Step 5 was to determine, for each priority nutrient, if an increased intake might be beneficial to address diet-related disease. For each priority nutrient, per each demographic group, the mean intake, the level of the suggested revised requirement, and the minimum dose associated with an effect on a health priority were expressed as a proportion of the Recommended Dietary Allowance (RDA) or AI for that nutrient (proportional intake), with the RDA/AI defined as being equal to 1. The RDA was used instead of the EAR to reflect the intake that is considered to be sufficient to meet the requirements of nearly all (97–98%) of the population [26]. For beneficial nutrients where the minimum dose was greater than the UL for a nutrient, the UL was taken to be the minimum dose given the established risks associated with exceeding the UL. These risks and limitations associated with the collected data were noted. For demographic groups comprising more than one age and/or sex group (as defined by national reference intakes or guidelines [26,27,28,29]), the RDA reflecting the greatest need was used to represent the demographic group to capture the highest level of inadequacy. If a nutrient was associated with multiple health priorities for a demographic, with a variation in the minimum dose across health priorities, the mean minimum dose was calculated and used. Similarly, if there were multiple suggestions for an increased need, the mean suggested level of need was used. If there were no data for a specific characteristic (for example, no level of inadequacy identified), the RDA or AI, as appropriate, was assumed to be applicable. The proportional intake for a population-level priority nutrient was calculated as the mean proportional intake ±SD for all demographic groups. Proportional intakes per each of the mean intake, level of suggested revised requirement, and minimum dose associated with an effect on a health priority were compared to assess the suitability of current RDIs to address diet-related diseases.

## 3. Results

### 3.1. Identification of Demographic Groups (Step 1)

A total of eight demographic groups were selected for this research: children (males and females) 4 to 8 years; adolescent males 9–18 years; adolescent females 9–18 years; adult males 19–70 years; adult females 19–50 years; pregnancy and lactation 19–50 years; menopause and post-menopause 51–70 years; and older adults (males and females) >70 years. Children under 4 years of age were excluded. Demographic groups were based predominantly on the age/sex groups defined by the Institute of Medicine (IOM) [26,36,37], with the collapse of some of these to produce larger demographic groupings: Male and female children were grouped together due to having similar intake needs for the majority of nutrients and an absence of major hormonal changes; Adolescent age groups of 9–13 years and 14–18 years were collapsed into one group (for each of males and females) due to having similar nutrient needs and hormonal changes appearing before the age of 13 in many individuals [40]. Adult male age groups of 19–30, 31–50, and 51–70 years were collapsed due to having similar nutrient requirements. Female demographic groups were defined based on the major life stages of fertility, pregnancy and lactation, and menopause and post-menopause. Male and female older adults greater than 70 years were collapsed due to an absence of menstrual fluctuations in women by this age.

### 3.2. Selection of Health Priorities (Step 2)

The final health priorities selected for each demographic group, including the health outcomes covered by each major health priority, are shown in Table 3.

Many health priorities featured across multiple demographic groups, such as cardiovascular health, metabolic health, cancer, mood, and mental health. Most demographic groups were characterized by one or more specific health priorities, such as fetal development for pregnancy and lactation, and fertility for adult females aged 19–50 years. For some health priorities, specificity was found within the included health outcomes; for example, while children and male and female adolescents shared a ‘growth and development’ health priority, pubertal development was included for male and female adolescents, while neurodevelopmental disorders were present for children. The health priority of ‘independence’ for older adults was constructed based on there being many aging-related health outcomes that influenced the ability of an individual to remain living independently, including physical mobility (such as muscle mass and osteoarthritis), resilience-related outcomes (such as frailty and risk of falls), and cognitive function.

### 3.3. Identification of Nutrients (Step 3)

A total of 21 nutrients (out of 24; 87.5%) were identified to have inadequate intake (N = 21), evidence for increased needs (N = 7), or an association with one or more health priorities (N = 14, 58%). Three nutrients showed beneficial and adverse associations (protein, folate, and omega-3 fatty acids), and one nutrient (copper) showed adverse associations only (cardiovascular health). The identified nutrients are summarized for each demographic group in Table 4, with additional detail provided in Supplementary Tables S2–S4.

Inadequate intakes were prominent across the demographic groups, with adolescent females showing the greatest number of inadequacies (N = 19), followed by pregnancy and lactation (N = 16). The demographic group with the lowest number of inadequate nutrients was children (N = 8). Some nutrients had very high levels of inadequacy (over 90% not meeting the EAR or AI) across all demographic groups, including dietary fiber, vitamin D, and choline (Supplementary Table S2). No nutrients were identified as being of concern, where intake was above the UL for more than 20% of a demographic group.

Nutrients with evidence for increased needs were identified for all demographic groups (Supplementary Table S3). In particular, there was evidence for increased protein and magnesium requirements across all demographic groups, and for zinc in all demographic groups except children. Some nutrients were suggested to have an increased need specific to one demographic group, such as vitamin D for menopause and post-menopause, and vitamin B12 for older adults. No evidence suggesting decreased needs for any nutrient for any demographic group with respect to the relevant DRIs was identified.

Over half of the included nutrients were associated with at least one health priority, with select nutrients showing associations with multiple health priorities across and within demographic groups (Supplementary Table S4). For example, vitamin D was beneficial for growth and development and respiratory health for both children and adolescents, metabolic health for all demographic groups, mood and mental health for all demographic groups except older adults, cardiovascular health for all demographic groups except children and adolescents, and cancer for all demographic groups except children. Significant associations were also found for dietary fiber, folate, and omega-3 fatty acids. Adverse associations were found for folate (increased risk for gestational diabetes mellitus [48] in pregnancy and lactation, and ADHD children and adolescents have higher blood folate levels [49]), omega-3 fatty acids (increased risk of atrial fibrillation with supplementation [50], and high doses of vitamin D (increased risk of falls and hip fracture in older adults [51,52]). Higher vs. lower protein intake was found to increase the risk of early menarche in adolescent females [53], and elevated blood copper levels were associated with an increased risk of stroke, coronary artery disease mortality, and CVD mortality in adults [54].

**Figure 2:**
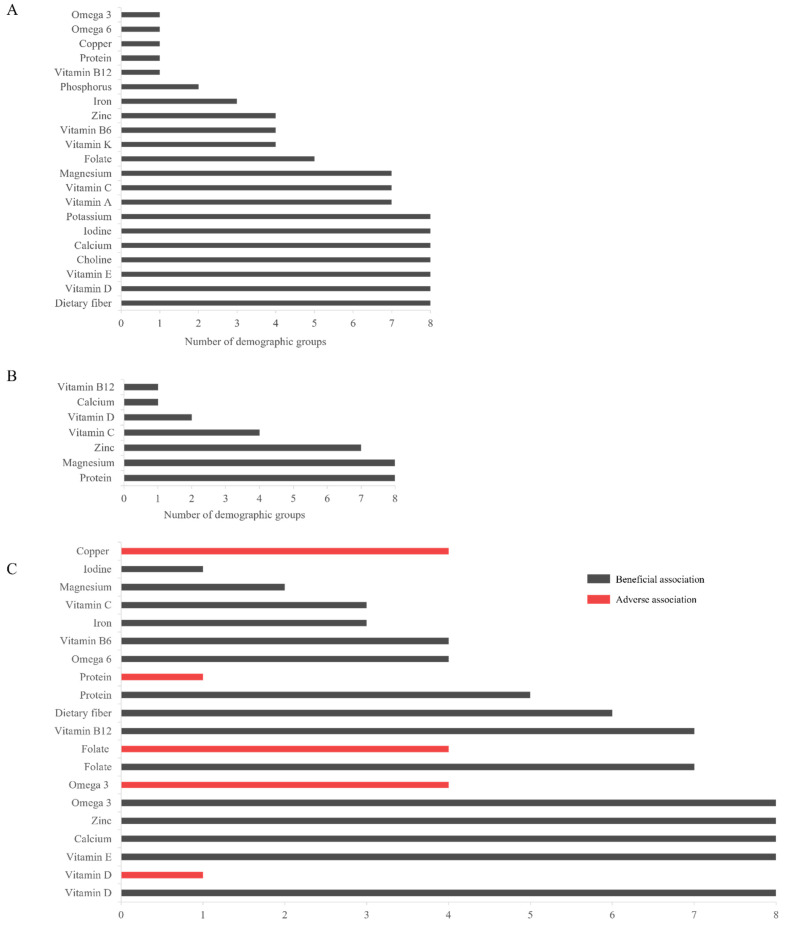
The number of demographic groups with each of (**A**) inadequate intake; (**B**) increased needs; and (**C**) association with health priorities, for each identified nutrient. Both beneficial (gray bars) and adverse (red bars) health associations are presented for each applicable nutrient. Abbreviations: omega-3, omega-3 fatty acids; omega-6, and omega-6 fatty acids.

Figure 2 depicts the number of demographic groups with inadequate intake (Figure 2A), increased needs (Figure 2A), and association with a health priority, for each nutrient. Seven nutrients had inadequate intake across all demographic groups: dietary fiber, vitamin D, vitamin E, choline, calcium, iodine, and potassium; some nutrients (such as protein and vitamin B12) were consumed inadequately in one demographic group. Increased needs were most prevalent for protein and magnesium, with a suggested increase in the DRI for all demographic groups. Similarly, the health of all demographic groups was found to benefit from vitamin D, vitamin E, calcium, and zinc. While omega-3 fatty acids and folate showed adverse health associations across four demographic groups, beneficial associations were present in more demographic groups (eight and six, respectively). Copper had only adverse associations in half of the demographic groups (all adult groups except pregnancy and lactation, Supplementary Table S4).

### 3.4. Prioritization of Nutrients (Step 4)

**Figure 3:**
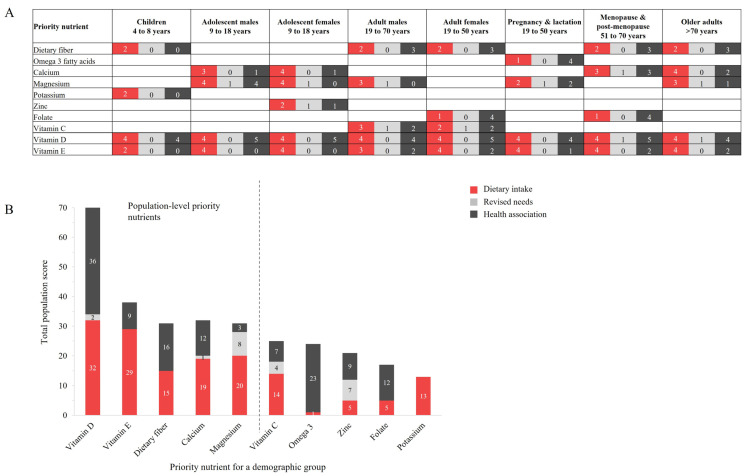
The priority nutrients for (**A**) each demographic group; and (**B**) the total population, including sub-scores for each of dietary intake (red), revised needs (light gray), and association with health priorities (dark gray). The highest priority nutrients for the overall population (left of dashed line, (**B**) are vitamin D, vitamin E, dietary fiber, calcium, and magnesium. Abbreviations: Omega-3, omega-3 fatty acids.

The highest priority nutrients by demographic group, and for the overall population, are shown in Figure 3. Sub-scores for dietary intake (red), revised needs (light gray), and health association (dark gray) are also presented. All priority nutrients were beneficial; no priority nutrients of concern were identified. A heatmap as a visual representation of nutrient scoring and prioritization across the demographic groups is provided in Supplementary Figure S1.

Ten nutrients emerged as a priority to address malnutrition and diet-related disease in at least one demographic group (Figure 3A); these were dietary fiber, omega-3 fatty acids, calcium, magnesium, potassium, zinc, folate, vitamin C, vitamin D, and vitamin E. Vitamins D and E were priority nutrients for all demographic groups, while potassium (children), zinc (adolescent females), and omega-3 fatty acids (pregnancy and lactation) were priority nutrients for one demographic group only. Sub-scores for the majority of priority nutrients were limited to dietary intakes and health association, with some priority nutrients achieving priority status solely due to inadequate dietary intake, such as dietary fiber, potassium, and vitamin E for children.

The highest priority nutrients across the overall US population were dietary fiber, vitamin D, vitamin E, calcium, and magnesium (Figure 3B), with vitamin D having the highest total priority score at 71 out of 80, almost double the next highest priority nutrient (vitamin E, with 38 points) and more than double that of other priority nutrients. Vitamin D showed high levels of both dietary inadequacy and beneficial health priority associations. This pattern was also reflected by dietary fiber, while vitamin E, calcium, and magnesium had scores that were predominated by dietary inadequacy.

### 3.5. Assessment of Required Intake to Address Diet-Related Disease (Step 5)

Table 5 summarizes the magnitude of dietary intake, suggested increased need, and dose range associated with beneficial effects on each health priority, for each priority nutrient in each demographic group. The proportional intake (PI) of each, relative to the RDA or AI (as applicable), is also shown. For most priority nutrients, dietary intake had a PI less than one (reflecting inadequacy), while the mean minimum dose associated with beneficial effects on a health priority was greater than one, reflecting that an increased daily intake relative to the DRI may be required to address diet-related disease. The exception to this was dietary fiber, for which a modest increase in daily intake was found to impart significant benefits for multiple health priorities, including metabolic health (at least 3 g/day [15]), cancer (10 g/day [55]), and cardiovascular health (at least 3 g/day [15,55,56]); the PI range was 0.6–0.99 (representing a total fiber intake of 60–99% of the AI) across applicable demographic groups.

**Figure 4:**
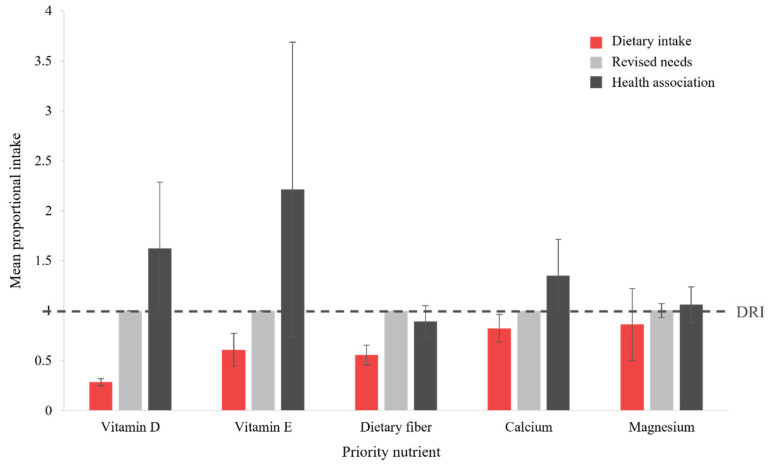
Comparison to the DRI (RDA or AI, as applicable, dashed line) for each population-level priority nutrient, for dietary intake (red bars), suggested level of revised needs (light gray bars), and minimum dose showing an association with a health priority (dark gray bars). Columns represent the mean proportional intake across all demographic groups, with error bars indicating the associated SD. Abbreviations: AI, Adequate Intake; DRI, Dietary Reference Intake; RDA, Recommended Dietary Allowance.

Integration of proportional intake values across the demographic groups for each of the population-level priority nutrients is depicted graphically in Figure 4. Comparison of mean PI values for dietary intake (red bars), level of revised needs (light gray bars), and minimum dose associated with a health priority (dark gray bars) shows that the current DRI (standardized, dashed line) for dietary fiber and magnesium aligns with the minimum dose found to address diet-related disease. For these nutrients, increased dietary intake to meet the DRI is required to address the health priorities of each relevant demographic group. While variability is high (large error bars), an increased intake, relative to the DRI, of vitamin D, vitamin E, and calcium may provide additional benefit in reducing the risk of and/or preventing diet-related disease.

## 4. Discussion

This research provides an evidence-based, holistic approach to the prioritization of nutrients that can be targeted to address both malnutrition and diet-related disease in the US. These priority nutrients have been identified across demographic groups and for the overall population, enabling both demographic-specific and nationwide recommendations that can help to close these nutrient gaps. Nutrient inadequacies were highly prevalent across all demographic groups, indicating a key gap in nutrient sufficiency, even within a developed nation. While ten nutrients were found to be the highest priority across the demographic groups, five nutrients were identified to be of utmost importance across the overall American population, showing both a high level of inadequate intake and multiple associations with health priorities. These priority nutrients were vitamin D, vitamin E, dietary fiber, calcium, and magnesium. Vitamin D and E were priority nutrients for every demographic group. A comparison of current and recommended intakes with intakes showing beneficial associations with major health priorities indicated that increased dietary targets for vitamin D, vitamin E, and calcium may be beneficial. Initiatives that increase the intake of priority nutrients to address malnutrition and diet-related disease in the US are needed. Recommendations can further be tailored to each demographic group by health professionals and governing bodies via food-based guidelines.

It is well-established that poor diet is a leading cause of mortality and morbidity related to chronic diseases in the US, such as T2D, CVD, obesity, musculoskeletal disorders, and some cancers [1,11]. Consistent with these data, metabolic health, cardiovascular health, and cancer were found to be major health priorities across almost all demographic groups in the current research, and multiple nutrient-health associations for these health outcomes were identified. However, the novel contribution of the research presented here is that many of the nutrients found to confer protective effects for diet-related disease were also consumed inadequately: the proportion of the population with an intake less than the EAR was over 97% for some nutrients. Thus, micronutrient inadequacies, particularly of nutrients that play a role in the prevention of chronic disease, are likely to be a critical factor in improving diet-related health in the US. Preventative approaches have been highlighted as a key priority for all stakeholders involved in public health, including physicians, nurses, hospital systems, policy-makers, health insurance companies, patients and their families, and advocacy groups [1]. Hence, this research can assist stakeholders in the prioritization of nutrients as part of a preventative solution to both nationwide malnutrition and diet-related disease rates: vitamin D, vitamin E, calcium, magnesium, and dietary fiber. Of these, calcium, dietary fiber, and vitamin D are considered to be nutrients of public health concern for the general US population because low intakes are associated with health concerns [29], adding weight to their priority status.

While the term ‘malnutrition’ has long been associated with protein-energy and micronutrient deficiency in developing countries, particularly for children and pregnant women [177], it is clear that nutrient inadequacies are prominent across the lifespan in developed countries such as AUS, NZ [12], and the US. Similarly, a recent analysis of global micronutrient intakes reported high rates of inadequacy for multiple micronutrients, especially (but not limited to) vitamin A, iodine, and calcium across the lifespan and regardless of a country’s economic status [4]. Although frank deficiencies such as goiter (iodine) and night blindness (vitamin A) are unlikely in developed countries, inadequate nutrient intake is a risk factor for deficiency [4], and is suggestive of dietary patterns that are unable to optimally support health. The World Health Organization defines malnutrition as deficiencies, excesses, or imbalances in a person’s intake of energy and/or nutrients [178]; thus, both micronutrient inadequacy and excess consumption of nutrients of concern (added sugars, sodium, and saturated fat) contribute to malnutrition in the US. It is possible that diet-related diseases are an overt symptom of malnutrition. In this study, 21 of the 24 assessed nutrients (87.5%) were consumed below recommended levels (at least 20% or 50% of the population consuming less than the EAR or AI, respectively) in one or more demographic groups. While seven nutrients showed inadequacy across all demographic groups (vitamin D, vitamin E, calcium, choline, iodine, magnesium, potassium, and dietary fiber), a set of inadequacies characterized each demographic group, indicating that targeted recommendations across the lifespan may also be useful. For example, it is concerning that adolescent females had the highest number of inadequate nutrients (N = 19), a life stage where development remains a key health priority. Similarly, there were 16 inadequate nutrients among the pregnancy and lactation demographic group. This highlights a need for increased monitoring and understanding of the nutritional status of this vulnerable population group, particularly as the data were collected primarily from the scientific literature, given that NHANES does not report on pregnancy and lactation. While children had the lowest number of inadequate nutrients (N = 8), this is a key demographic group for early preventative intervention, where addressing these insufficiencies supports health later in life and can help to establish healthy dietary patterns. The findings support the assertion that nutrient inadequacy is a significant factor underpinning the relationship between diet and chronic disease in the US population.

It is important to recognize that, in addition to nutrient requirements, health priorities and nutrient-health associations also vary by age and sex, resulting in a unique set of priority nutrients for each of the eight demographic groups. Nutritional targets that can address the specific nutrition and diet-related health priorities of each life stage are important. While many priority nutrients were common throughout the demographic groups, some showed specificity to one group only, including potassium (children), zinc (adolescent females), and omega-3 fatty acids (pregnancy and lactation). Similarly, some nutrients showed adverse associations with specific demographic groups, such as folate for pregnancy/lactation and children/adolescents, vitamin D for older adults, and omega-3 fatty acids for all adults. It is important to note that these adverse associations were limited to high intakes and/or blood levels of each nutrient or its supplemental form, with intake levels much higher than the observed average intakes for each relevant demographic group [28]. For folate, adverse associations were primarily for the highest levels of red blood cell or serum folate, with limited information regarding actual folate intake [48,49]. Personalized nutrition and ‘food as medicine’ approaches have been recognized as playing an important role in addressing the diet-related disease status of the US [11]. The prioritization of foods that contain priority nutrients can be utilized by health professionals, including medical practitioners, to provide nutrition recommendations that are more specific to life stages. These priority nutrients are valuable for policy development and government-led health initiatives, to ensure tangible outcomes and optimize return on investment, as well as the prioritization of research funding and product re-formulation and development. These findings may also support international collaboration efforts between countries, given the similarity in priority nutrients across countries. AUS/NZ [12] had four priority nutrients in common with the USA: vitamin D, calcium, magnesium, and dietary fiber. A recent analysis of the global prevalence of micronutrient inadequacy found high rates for both calcium (66%, 5 billion people) and magnesium (31%, 2.4 billion people) [4]; fiber and vitamin D were not included in this research. While focused research on additional countries is needed, the evidence suggests that key nutrients may be targeted and specified within international recommendations to have a global impact on health.

A key component of the nutrient prioritization is the translation of nutrient needs into dietary patterns [11], with a focus on the foods that can provide priority nutrients. For example, prioritizing recommendations for foods such as fatty fish, eggs, and UV-exposed mushrooms would assist with intakes for vitamin D. Dairy, including milk and milk products provide the priority nutrients calcium, magnesium, and vitamin D (via fortification), and vegetables, grains, nuts, and seeds, provide magnesium, dietary fiber, and vitamin E [29,179,180]. Prioritizing foods rich in priority nutrients has increased importance given the rise in consumption of foods high in nutrients of concern (added sugars, saturated fat, and sodium), alongside a decline in grains, lean protein, and dairy in the US [181,182], particularly post the COVID pandemic [183]. For some priority nutrients, this may be exacerbated by recommendations to limit the consumption of animal food sources and an increasing movement towards a plant-based diet, as well as differences in nutrient bioavailability between animal and plant foods [26,184]. Thus, in addition to efforts to reduce the intake of foods high in nutrients of concern, an increased focus on the consumption of foods rich in priority nutrients is necessary. To support priority nutrient intake, supplementation or food fortification may also be required for some demographic groups, for example, older adults who find it difficult to consume an adequate intake of food overall, or during pregnancy and lactation. While the intent of this analysis was not to assess the suitability of current DRIs, intakes greater than the currently recommended for calcium, vitamin D, and vitamin E were found to be of additional health benefit. There was a high degree of variability at the population level, due to specific findings per each age/sex grouping, highlighting the need for the provision of demographic-level recommendations and guidance. For example, high supplemental doses of vitamin D may have an adverse effect on older adults. These findings increase the importance of a focus on foods providing these nutrients for the purpose of diet-related disease prevention.

This research contains both strengths and limitations. The applicability of the methodology to both the US population and each demographic group specifically allows for priority nutrient recommendations to be made at both the national level and for specific life stages. The stepwise methodology lends itself to consistency, for comparison between countries and different age/sex groupings, as well as modification as appropriate to any country or demographic group. Further research employing this methodology can answer key questions about the optimal nutritional approach to support health in a given situation; for example, the approach could focus specifically on the priority nutrients to address digestive issues in athletes, or depression in middle-aged males, with the quality of the findings depending primarily on the breadth of scope and available data. While survey data used for the assessment of dietary nutrient intake were the most recently available [27], these data were pre-COVID and may not accurately reflect the current post-COVID situation. In addition, only the highest priority health outcomes were included in the assessment, preventing the inclusion of secondary diet-related health outcomes that may be important for sub-populations of some demographic groups.

Finally, due to the system’s level nature of research, covering multiple nutrients, health associations, and demographic groups, the search carried out for nutrient-health associations was targeted rather than systematic, and thus limited to the research identified at the time of investigation. Similarly, there was no formal assessment of study quality or certainty of evidence, limiting the ability to make direct recommendations regarding the inclusion and dose of priority nutrients within nutrition guidelines. The methodology can be reproduced with increased detail for one or more demographic groups or sub-groups, allowing for additional health outcomes and/or a systematic approach to be included in the analysis. This would be the recommended approach, including a formal assessment of evidence quality (such as AMSTAR) and certainty (such as GRADE) prior to providing specific population guidance. Further iterations of the methodology can also focus on subtypes of nutrients based on dietary source, which were not addressed here; for example, animal vs. plant protein and vegetable vs. cereal fiber. Regardless of the multiple refinements that could be made, the analysis presented provides an overarching assessment of the priority nutrients for the US population, an important step in addressing modifiable causes of morbidity and mortality.

## 5. Conclusions

This research identifies ten key nutrients showing both inadequate intakes and a role in the prevention of diet-related disease in the US population, with five nutrients common across all demographic groups. These priority nutrients can be targeted for increased consumption as part of the strategy to improve public health outcomes. This includes priority nutrients in American nutrition policies and dietary guidelines, as well as diet-related initiatives and programs are warranted. A framework that can be adapted to support these initiatives has been presented. Trends that exclude foods rich in priority foods may contribute to and/or potentially exacerbate existing inadequacies.

## Figures and Tables

**Table 1:** Nutrients included in the assessment.

Nutrient Type	Nutrients Included in the Priority Assessment
Macronutrients	Protein, dietary fiber, *n*-6 (linoleic) fatty acids, *n*-3 (ALA, DHA, EPA) fatty acids.
Vitamins	Vitamin A, vitamin C, vitamin D, vitamin E, vitamin K, thiamine, riboflavin, niacin, vitamin B6, folate, B12, choline.
Minerals	Calcium, copper, iodine, iron, magnesium, phosphorus, potassium, selenium, zinc.

ALA, alpha-linolenic acid; DHA, docosahexaenoic acid; EPA, eicosapentaenoic acid; *n*-3, omega-3; *n*-6, omega-6.

**Table 2:** Quantitative scoring matrix for nutrient prioritization.

Scoring Criteria	Beneficial Nutrient Scoring	Score	Nutrient of Concern Scoring	Score
Dietary intake score	No inadequacy	0	No excess	0
	≥20 to <40 population less than EAR *OR* ≥50 to <75% population less than AI *OR* mean intake < EAR or AI	1	≥20 to <40 population greater than the UL	−1
	≥40 to <60 population less than EAR *OR* ≥75 population less than AI	2	≥40 to <60 population greater than the UL	−2
	≥60 to <80 population less than EAR	3	≥60 to <80 population greater than the UL	−3
	≥80 population less than EAR	4	≥80 population greater than the UL	−4
	Sub-score dietary intake	Beneficial nutrient or nutrient of concern baseline score		
Revised needs score	No increased need	0	No decreased need	0
	Increased need identified	1	Decreased need identified	−1
	Sub-score revised needs	Increased need or decreased need score		
Health association score	No beneficial association	0	No adverse association	0
	Beneficial association with 1 health priority	1	Adverse association with 1 health priority	−1
	Beneficial association with 2 to 5 health priorities	Σ_2_^5^	Adverse association with 2 to 5 health priorities	−Σ_2_^5^
	Sub-score health association	Beneficial association score minus adverse association score		
TOTAL PRIORITY SCORE	Sub-score dietary intake + Sub-score revised needs + Sub-score health association			

AI, Adequate Intake; EAR, Estimated Average Requirement; UL, Tolerable Upper Intake Level; Σ_2_^5^, sum of number of associations between 2 and 5.

**Table 3:** Health priorities and included health outcomes for each demographic group.

Demographic	Health Priority	Health Outcomes Included
Children 4–8 years	Growth and development	Bone health, cognitive development, muscle development, and neurodevelopmental disorders
	Respiratory health	Respiratory allergies, asthma, and respiratory infections
	Metabolic health	Obesity and bodyweight-related markers, body composition, blood glucose control, insulin resistance
	Mood and mental health	Mood disorders, depression, anxiety
Adolescent males 9–18 years	Growth and development	Bone health, cognitive development, muscle development, and pubertal development
	Mood and mental health	*Idem.*
	Metabolic health	*Idem.*
	Respiratory health	*Idem.*
	Cancer	Total cancer, individual cancers (all types), and cancer-related mortality
Adolescent females 9–18 years	Growth and development	Bone health, cognitive development, muscle development, and pubertal development
	Mood and mental health	*Idem.*
	Metabolic health	*Idem.*
	Respiratory health	*Idem.*
	Cancer	*Idem.*
Adult males 19–70 years	Metabolic health	*Idem.*
	Cardiovascular health	Risk and risk factors (including cholesterol, triglycerides) for cardiovascular diseases (including stroke), and cardiovascular disease-related mortality
	Cancer	*Idem.*
	Mood and mental health	*Idem.*
Adult females 19–50 years	Fertility	Fertility-related markers, including in vitro fertilization rate, ovarian reserve, pregnancy rate, implantation, and miscarriage
	Cardiovascular health	*Idem.*
	Cancer	*Idem.*
	Mood and mental health	*Idem.*
	Metabolic health	*Idem.*
Pregnancy and lactation 19–50 years	Fetal development	Markers for healthy development include birth size, weight, and length, premature birth, labor induction, live birth rate, neurodevelopment, and health during childhood
	Cardiovascular health	*Idem.*
	Metabolic health	*Idem.*
	Mood and mental health	*Idem.*
	Infectious immunity	Infection and infectious disease incidence and risk
Menopause and post-menopause 51–70 years	Cardiovascular health	*Idem.*
	Cancer	*Idem.*
	Bone density	Bone mineral density, osteoporosis, fractures, and fracture risk
	Metabolic health	*Idem.*
	Mood and mental health	*Idem.*
Older adults >70 years	Cardiovascular health	*Idem.*
	Cancer	*Idem.*
	Metabolic health	*Idem.*
	Infectious immunity	*Idem.*
	Independence	Physical and cognitive independence, including incidence and risk of sarcopenia, frailty, osteoporosis, osteoarthritis, cognitive function/decline, fractures, and falls

*Idem*., as previously stated for the health priority.

**Table 4:** Nutrients identified as either having inadequate intakes ^1^, increased needs ^2^, or association with health priorities ^3^ by demographic group.

Demographic	Inadequate Intake ^1^	Increased Needs ^2^	Health Priority Association ^3^
Children 4–8 years	Dietary fiber, vitamin D, vitamin E, vitamin K, choline, calcium, iodine, potassium	Protein, magnesium	**Beneficial** Calcium, iron, zinc, vitamin B12, vitamin D, omega-3 fatty acids **Adverse** Folate
Adolescent males 9–18 years	Dietary fiber, vitamin A, vitamin C, vitamin D, vitamin E, choline, calcium, iodine, magnesium, phosphorus, potassium	Zinc, protein, magnesium	**Beneficial** Calcium, vitamin D, iron, zinc, vitamin B12, and omega-3 fatty acids **Adverse** Folate
Adolescent females 9–18 years	Protein, dietary fiber, vitamin A, vitamin C, vitamin D, vitamin E, vitamin K, vitamin B6, folate, vitamin B12, choline, calcium, copper, iodine, iron, magnesium, phosphorus, zinc, potassium	Zinc, protein, magnesium	**Beneficial** Calcium, vitamin D, iron, zinc, vitamin B12, dietary fiber, omega-3 fatty acids **Adverse** Protein, folate
Adult males 19–70 years	Dietary fiber, vitamin A, vitamin C, vitamin D, vitamin E, vitamin K, choline, calcium, iodine, magnesium, zinc, potassium	Zinc, protein, magnesium, and vitamin C	**Beneficial** Vitamin D, omega-3 fatty acids, vitamin C, vitamin E, folate, zinc, dietary fiber, calcium, omega-6 fatty acids, protein, vitamin B12, and vitamin B6 **Adverse** Omega-3 fatty acids, copper
Adult females 19–50 years	Dietary fiber, vitamin A, vitamin C, vitamin D, vitamin E, folate, choline, calcium, iodine, iron, magnesium, potassium	Zinc, protein, magnesium, and vitamin C	**Beneficial** Vitamin D, omega-3 fatty acids, vitamin C, vitamin E, folate, zinc, dietary fiber, calcium, omega-6 fatty acids, protein, vitamin B12, and vitamin B6 **Adverse** Omega-3 fatty acids, copper
Pregnancy and lactation 19–50 years	Dietary fiber, linoleic acid, alpha-linolenic acid, vitamin A, vitamin C, vitamin D, vitamin E, vitamin B6, folate, choline, calcium, iodine, iron, magnesium, zinc, potassium	Zinc, protein, magnesium	**Beneficial** Vitamin D, omega-3 fatty acids, vitamin E, dietary fiber, folate, magnesium, zinc, calcium, iodine **Adverse** Folate
Menopause and postmenopause 51–70 years	Dietary fiber, vitamin A, vitamin C, vitamin D, vitamin E, vitamin B6, folate, choline, calcium, iodine, magnesium, potassium	Zinc, protein, magnesium, vitamin C, vitamin D, calcium	**Beneficial** Vitamin D, omega-3 fatty acids, vitamin C, vitamin E, folate, zinc, dietary fiber, calcium, omega-6 fatty acids, protein, vitamin B12, and vitamin B6 **Adverse** Omega-3 fatty acids, copper
Older adults >70 years	Dietary fiber, vitamin A, vitamin C, vitamin D, vitamin E, vitamin K, vitamin B6, folate, choline, calcium, iodine, magnesium, zinc, potassium	Zinc, protein, magnesium, vitamin C, vitamin D, vitamin B12	**Beneficial** Dietary fiber, vitamin D, omega-3 fatty acids, vitamin C, vitamin E, calcium, omega-6 fatty acids, folate, protein, zinc, vitamin B12, vitamin B6, magnesium **Adverse** Omega-3 fatty acids, copper

^1^ Intake data was based on NHANES 2017 to pre-pandemic reports [27] for all demographic groups except pregnancy, where data was sourced from older NHANES intake reports [41,42] as well as supporting information from the scientific literature [43,44,45,46,47]. No nutrients were identified as being consumed in excess. Details are provided in Supplementary Table S2. ^2^ Consensus was identified in the scientific literature. Details and references are provided in Supplementary Table S3. No nutrients were identified as being consumed in excess. ^3^ Based on consistent evidence from SLRs of RCTs and prospective cohort studies. Details and references are provided in Supplementary Table S4. Both beneficial and adverse relationships are shown. NHANES, National Health and Nutrition Examination Survey.

**Table 5:** Summary of intake ^1^, suggested increase ^2^, dose range associated with a beneficial effect on a health priority ^3^, and estimation of proportional intake ^4^ for each priority nutrient for each demographic group.

Priority Nutrient	DRI ^5^	Intake		Increased Need		Beneficial Association with a Health Priority	
		Mean Intake ^1,6^	PI ^4^	Suggested Increase ^2^	PI ^4^	Dose Range Associated with One or More Health Priorities ^3^	PI ^4^
Children 4–8 years							
Dietary fiber	AI: 25 g/day	13.2–13.8 g/day	0.54	NA	1	NA	1
Vitamin D	RDA: 15 µg/day	4.7–5.5 µg/day	0.34	NA	1	132–7000 IU/day [18,57,58,59,60,61,62,63,64,65]	1.41
Vitamin E	RDA: 7 mg/day	6.2–7.2 mg/day	0.96	NA	1	NA	1
Potassium	AI: 2300 mg/day	1920–2082 mg/day	0.87	NA	1	NA	1
Adolescent males 9–18 years							
Vitamin D	RDA: 15 µg/day	4.7–5.4 µg /day	0.31	NA	1	132–7000 IU/day; [18,57,58,59,61,62,63,64,65,66,67] H vs. L blood status [19,68,69,70]	1.24
Vitamin E	RDA: 15 mg/day	8.6–9 mg/day	0.57	NA	1	NA	1
Calcium	RDA: 1300 mg/day	1036–1081 mg/day	0.81	NA	1	460–850 mg/day [16,71]	1.17
Magnesium	RDA: 410 mg/day	247–276 mg/day	0.64	Not provided [72]	1	NA	1
Adolescent females 9–18 years							
Vitamin D	RDA: 15 µg/day	3.2–4.8 µg/day	0.27	NA	1	132–7000 IU/day; [18,57,58,59,61,62,63,64,65,66,67] H vs. L blood status [19,68,69,70]	1.2
Vitamin E	RDA: 15 mg/day	7.3–8.7 mg/day	0.53	NA	1	NA	1
Calcium	RDA: 1300 mg/day	811–987 mg/day	0.69	NA	1	460–850 mg/day [16,71]	1.05
Magnesium	RDA: 360 mg/day	218–246 mg/day	0.64	Not provided [72]	1	NA	1
Zinc	RDA: 9 mg/day	7.7–9.6 mg/day	0.96	10% increase [73]	1.1	1.66–5.6 mg/day [74,75]	0.98
Adult males 19–70 years							
Dietary fiber	AI: 38 g/day	16.1–18.8 g/day	0.46	NA	1	3–35 g/day; H vs. L intake; increase of 10–15 g/day [13,15,55,56,76,77,78,79,80,81]	0.6
Vitamin C	RDA: 90 mg/day	68.7–87.5 mg/day	0.87	>75–200 mg/day [82,83]	1.53	100–3000 mg/day; H vs. L intake; H vs. low blood levels [84,85,86]	3.75
Vitamin D	RDA: 15 µg/day	4.2–5.2 µg/day	0.31	NA	1	1000–14,000 IU/day; H vs. L blood levels; H vs. L intake [19,21,50,62,65,68,69,70,87,88,89,90,91,92,93,94,95,96,97,98,99,100,101,102,103,104,105,106,107,108,109,110,111]	2.48
Vitamin E	RDA: 15 mg/day	9.3–10.8 mg/day	0.67	NA	1	54–1200 mg/day; increase of 10 mg/day; H vs. L intake; H vs. L blood levels) [112,113]	2.67
Magnesium	RDA: 420 mg/day	306–353 mg/day	0.78	Not provided [72]	1	NA	1
Adult females 19–50 years							
Dietary fiber	AI: 25 g/day	14.3–15.2 g/day	0.59	NA	1	3–35 g/day; H vs. L intake; increase of 10–15 g/day [13,15,55,56,76,77,78,79,80,81]	0.8
Vitamin D	RDA: 15 µg/day	3.5–3.8 µg/day	0.24	NA	1	1000–14,000 IU/day; H vs. L blood levels; H vs. L intake [19,21,62,65,68,69,70,87,88,89,90,91,93,94,95,96,114,115,116,117,118,119,120,121,122,123,124]	2.58
Vitamin E	RDA: 15 mg/day	8.7 mg/day	0.58	NA	1	54–1200 mg/day; increase of 10 mg/day; H vs. L intake; H vs. L blood levels) [112,113]	2.67
Vitamin C	RDA: 75 mg/day	66.8–71.5 mg/day	0.92	>75–200 mg/day [82,83]	1.83	100–3000 mg/day; H vs. L intake; H vs. low blood levels [84,85,86]	4.5
Folate	RDA: 400 µg/day	431–432 µg/day	1.08	NA	1	0.5 to 30 mg/day folic acid; 7.5 to 15 mg/day 5-MTHF; H vs. L intake; H vs. L blood levels [125,126,127,128,129,130,131]	2.5 ^7^
Pregnancy and lactation 19–50 years							
Vitamin D	RDA: 15 µg/day	3.6–5.5 µg/day	0.3	NA	1	400–7100 IU/day; S vs. D blood status; H vs. L blood status [62,122,132,133,134,135,136,137,138,139,140,141,142,143,144,145,146,147,148,149,150]	0.96
Magnesium	RDA: 360 mg/day	254–294 mg/day	0.76	Not provided [72]	1	150 mg Mg equivalent [151]	1.18
Omega-3 fatty acids	AI: 1.4 g/day (ALA)	1.29 g/day (ALA + DHA + EPA)	0.92	NA	1	200–3000 mg/day [152,153,154,155,156,157]	1.32
Vitamin E	RDA: 19 mg/day	6.3–7.8 mg/day	0.37	NA	1	7.9–400 mg/day [158]	1.42
Menopause and post-menopause 51–70 years							
Dietary fiber	AI: 21 g/day	15.5 g/day	0.74	NA	1	3–35 g/day; H vs. L intake; increase of 10–15 g/day [13,15,55,56,76,77,78,79,80,81]	0.99
Vitamin D	RDA: 15 µg/day	3.8 µg/day	0.25	Not provided [159]	1	400–14,000 IU/day; H vs. L intake; H vs. L blood levels [19,21,62,65,68,69,70,87,88,89,90,91,92,93,94,95,96,97,101,114,115,116,117,118,119,120,121,160,161,162,163,164]	2.12
Vitamin E	RDA: 15 mg/day	8.4 mg/day	0.56	NA	1	54–1200 mg/day; increase of 10 mg/day; H vs. L intake; H vs. L blood levels) [112,113]	2.67
Folate	RDA: 400 µg/day	400 µg/day	1	NA	1	0.5 to 30 mg/day folic acid; 7.5 to 15 mg/day 5-MTHF; H vs. L folate/folic acid intake [101,125,126,127,128,129,131]	2.5 ^7^
Calcium	RDA: 1200 mg/day	832 mg/day	0.69	NA	1	350–1200 mg/day [17,165,166,167]	1.4
Older adults > 70 years							
Dietary fiber	AI: 30 g/day	15.3–19.1 g/day	0.57	NA	1	3–35 g/day; H vs. L intake; increase of 10–15 g/day [13,15,55,56,76,77,78,79,80,81]	0.75
Vitamin D	RDA: 20 µg/day	4.3–5.9 µg/day	0.26	Not provided [159,168]	1	400–8000 IU/day; H vs. L blood levels; H vs. L intake; 0.25–1 µg/day prescription forms of vitamin D (active) [19,21,51,62,68,69,70,92,94,95,96,97,101,114,115,116,117,118,119,120,121,160,164,169,170,171,172,173,174,175]	1.62
Vitamin E	RDA: 15 mg/day	9.2–10.2 mg/day	0.61	NA	1	134–1200 mg/day; increase of 10 mg/day; H vs. L intake; H vs. L blood levels [113]	5.3
Calcium	RDA: 1200 mg/day	785–968 mg/day	0.73	NA	1	≥1000 mg/day; increase of 350 mg/day [165,166]	1.29
Magnesium	RDA: 420 mg/day	256–330 mg/day	0.7	Not provided [72]	1	H vs. L blood levels [176]	1

^1^ Intake data were sourced from NHANES 2017 to pre-pandemic reports [27] for all the demographic groups except pregnancy, where data were sourced from older NHANES intake reports [41,42] as well as supporting information from the scientific literature [43,44,45,46,47]. Additional details are provided in Supplementary Table S2. ^2^ Where consensus was identified in the scientific literature, references as listed within the table. Additional details are provided in Supplementary Table S3. ^3^ Based on consistent evidence from SLRs of RCTs and prospective cohort studies, references as listed within the table. Additional details are provided in Supplementary Table S4. ^4^ Proportional intake was estimated based on the ratio between the level of intake and RDA or AI (as applicable [26,36,37]) for each of dietary intake (mean dietary intake divided by maximum RDA or AI), suggested increase (mean suggested total intake divided by RDA or AI), and intake associated with a beneficial effect on a health priority (mean minimum dose divided by RDA or AI) for each for priority nutrient for each demographic group. ^5^ DRI represents the maximum (where multiple age/sex groupings are included) recommended EAR, RDA, or AI [26,36,37] for each demographic group. ^6^ Vitamin D values have been reported in µg/day to reflect the survey method of reporting. 1 µg/day is equivalent to 40 IU/day. ^7^ The minimum dose for folate exceeded the recommended UL for supplementation; the UL was used to calculate PI. AI, Adequate Intake; EAR, Estimated Average Requirement; RDA, Recommended Dietary Allowance; UL, Tolerable Upper Intake Level; DRI, Dietary Reference Intake; NHANES, National Health and Nutrition Examination Survey; PI, proportional intake; SLR, systematic literature review; RCT, randomized controlled trial; H, high; L, low; S, sufficient; D, deficient; IU, international units; NA, not applicable.

## Data Availability

The data presented in this study were derived from resources available in the public domain: https://www.ars.usda.gov/northeast-area/beltsville-md-bhnrc/beltsville-human-nutrition-research-center/food-surveys-research-group/docs/wweia-usual-intake-data-tables/. Data were accessed on 12 January 2023.

## Supplementary Materials

The following supporting information can be downloaded at: https://www.mdpi.com/article/10.3390/nu17121957/s1, Table S1. Search strategy for each of the nutrients and bioactives with each identified health priority; Table S2. Dietary intake data detailing inadequately consumed nutrients for each demographic group; Table S3. Summary of evidence identified for nutrients where an increased need over the DRI has been identified for each demographic group; Table S4. Summary of evidence identified for nutrients showing an association with one or more health priorities for each demographic group; Figure S1. Heatmap showing the level of inadequate intake (I), suggested increased need (IN), and number of associations with health priorities (HP) for each included nutrient within each demographic group. Nutrient scoring and therefore the degree of need for prioritization increases from green to dark red.

## Abbreviations

The following abbreviations are used in this manuscript:

AI	Adequate Intake
AUS	Australia
CDC	Centers for Disease Control and Prevention
CVD	cardiovascular disease
DHA	docosahexaenoic acid
EPA	eicosapentaenoic acid
EAR	Estimated Average Requirement
DRI	Dietary Reference Intake
IU	International units
NHANES	National Health and Nutrition Examination Survey
NRV	Nutrient Reference Value
NZ	New Zealand
RCT	randomized controlled trial
RDA	Recommended Dietary Allowance
SDT	Suggested Dietary Target
SLR	systematic literature review
T2D	type 2 diabetes
UL	Tolerable Upper Intake Level
US	United States of America
WWEIA	What We Eat in America
