# Supplementary material for: Identification of Priority Nutrients in the US: Targeting Malnutrition to Address Diet-Related Disease Across the Lifespan
Source: Nutrients. 2025 Jun 9;17(12):1957. doi: 10.3390/nu17121957 (PMC12196107; doi:10.3390/nu17121957)
Supplement: Supplementary file 1 [file nutrients-17-01957-s001.zip › nutrients-3653352-supplementary.pdf]

Supplementary Material

Supplementary Table S1. Search strategy for each of the nutrients and bioactives with each identified health priority.

**Component Of Search**	**Search Strategy**
Nutrients	((("omega 3"[Title/Abstract]) OR ("omega 6"[Title/Abstract])) OR ("dietary fibre"[Title/Abstract])) OR ("dietary fiber"[Title/Abstract])) OR (Docosapentaenoic[Title/Abstract])) OR (Eicosapentaenoic[Title/Abstract])) OR (Docosahexaenoic[Title/Abstract])) OR (DPA[Title/Abstract])) OR (EPA[Title/Abstract])) OR (DHA[Title/Abstract])) OR (linolenic[Title/Abstract])) OR (linoleic[Title/Abstract])) OR (protein[Title/Abstract])) OR (tocopherol[Title/Abstract])) OR (retinol[Title/Abstract])) OR (pyridoxine[Title/Abstract])) OR (cobalamin[Title/Abstract])) OR (niacin[Title/Abstract])) OR (riboflavin[Title/Abstract])) OR (thiamin[Title/Abstract])) OR (biotin[Title/Abstract])) OR (pantothenic[Title/Abstract])) OR ("folic acid"[Title/Abstract])) OR (folate[Title/Abstract])) OR (vitamin[Title/Abstract])) OR (zinc[Title/Abstract])) OR (sodium[Title/Abstract])) OR (potassium[Title/Abstract])) OR (phosphorus[Title/Abstract])) (magnesium[Title/Abstract])) OR (iodine[Title/Abstract])) OR (copper[Title/Abstract])) OR (Calcium[Title/Abstract])) OR ("dietary fiber"[MeSH Terms])) OR (fatty acids, unsaturated[MeSH Terms])) OR (fatty acids, omega 6[MeSH Terms])) OR (fatty acids, omega 3[MeSH Terms])) OR ("fatty acids, essential"[MeSH Terms])) OR ("diet therapy"[MeSH Terms])) OR ("phosphorus"[MeSH Terms])) OR ("trace elements"[MeSH Terms])) OR ("micronutrients"[MeSH Terms])) OR ("avitaminosis"[MeSH Terms])) OR ("avitaminosis"[MeSH Terms])) OR ("nutrients"[MeSH Terms]))
AND Study Types	((("meta analysis"[Publication Type]) OR ("guideline"[Publication Type])) OR ("systematic review"[Publication Type]))
AND Growth and development	(((((((((((((((((((((((((((((((("bone density"[MeSH Terms]) OR ("bone and bones"[MeSH Terms])) OR (bone diseases, metabolic[MeSH Terms])) OR ("bone density"[Title/Abstract])) OR ("fractures, bone"[MeSH Terms])) OR (fracture*[Title/Abstract])) OR (osteoporo*[Title/Abstract])) OR (osteopeni*[Title/Abstract])) OR ("cognition"[MeSH Terms])) OR ("intelligence"[MeSH Terms])) OR ("executive function"[MeSH Terms])) OR (brain[Title/Abstract])) OR (cognitive[Title/Abstract])) OR (cognition[Title/Abstract])) OR (IQ[Title/Abstract])) OR (intelligence[Title/Abstract])) OR ("musculoskeletal development"[MeSH Terms])) OR (skeletal[Title/Abstract])) OR (muscle[Title/Abstract])) OR ("puberty"[MeSH Terms])) OR ("genitalia"[MeSH Terms])) OR (puberty[Title/Abstract])) OR (skeletal[Title/Abstract])) OR (muscle[Title/Abstract])) OR ("sex organ"[Title/Abstract])) OR ("sexual organ"[Title/Abstract]) ("neurodevelopmental disorders"[MeSH Terms]) OR ("autistic disorder"[MeSH Terms])) OR ("autism spectrum disorder"[MeSH Terms])) OR ("attention deficit and disruptive behavior disorders"[MeSH Terms])) OR (ADHD[Title/Abstract])) OR (ASD[Title/Abstract]) OR (attention deficit

	[Title/Abstract])) OR (autism[Title/Abstract])) OR (neurodevelopment*[Title/Abstract])) OR (behaviour[Title/Abstract])) OR (behavior[Title/Abstract])) **AND** ((((((("child"[MeSH Terms]) OR (child*[Title/Abstract])) OR (pediatric*[Title/Abstract])) OR (paediatric*[Title/Abstract])) OR ("adolescent"[MeSH Terms]) OR (adolescen*[Title/Abstract]))) OR (adolescen*[Title/Abstract])) OR (teen*[Title/Abstract])
OR Mood and mental health	(((((((("mental health"[MeSH Terms]) OR ("anxiety disorders"[MeSH Terms])) OR ("disruptive, impulse control, and conduct disorders"[MeSH Terms])) OR (mood disorders[MeSH Terms])) OR (Depression[Title/Abstract])) OR (Anxiety[Title/Abstract])) OR (anxious[Title/Abstract])) OR (depressive[Title/Abstract])) OR ("mental health"[Title/Abstract]) **AND** (((((((("child"[MeSH Terms]) OR ("adolescent"[MeSH Terms])) OR (child*[Title/Abstract])) OR (teen*[Title/Abstract])) OR (adolescen*[Title/Abstract])) OR (pediatric*[Title/Abstract])) OR (paediatric*[Title/Abstract])) OR (adult*[Title/Abstract])) OR (menopaus*[Title/Abstract]))
OR Respiratory health	(((((("respiratory hypersensitivity"[MeSH Terms]) OR ("asthma"[MeSH Terms])) OR ("rhinitis, allergic"[MeSH Terms])) OR (“respiratory allergies” [Title/Abstract])) OR (rhinitis[Title/Abstract])) OR (asthma*[Title/Abstract]) **AND** ((((((("child"[MeSH Terms]) OR ("adolescent"[MeSH Terms])) OR (child*[Title/Abstract])) OR (teen*[Title/Abstract])) OR (adolescen*[Title/Abstract])) OR (pediatric*[Title/Abstract])) OR (paediatric*[Title/Abstract]))
OR Cancer	((((((("neoplasms"[MeSH Terms]) OR (cancer[Title/Abstract])) OR (tumour[Title/Abstract])) OR (tumor[Title/Abstract])) OR (lymphoma[Title/Abstract])) OR (myeloma[Title/Abstract])) OR (leukemia[Title/Abstract])) OR (oncolog*[Title/Abstract])
OR Cardiovascular health	(((((((((("heart disease risk factors"[MeSH Terms]) OR ("heart diseases"[MeSH Terms])) OR ("cardiovascular diseases"[MeSH Terms])) OR (CVD[Title/Abstract])) OR (CHD[Title/Abstract])) OR (IHD[Title/Abstract])) OR (cardiovascular[Title/Abstract])) OR (coronary[Title/Abstract])) OR ("heart disease"[Title/Abstract])) OR (stroke[Title/Abstract]) OR (ischem*[Title/Abstract]))
OR Metabolic health	(((((((((("obesity"[MeSH Terms]) OR ("metabolic syndrome"[MeSH Terms])) OR ("insulin resistance"[MeSH Terms])) OR ("diabetes mellitus, type 2"[MeSH Terms])) OR ("metabolic diseases"[MeSH Terms])) OR (T2DM[Title/Abstract])) OR (T2D[Title/Abstract])) OR (“type 2 diabetes”[Title/Abstract])) OR (“metabolic syndrome”[Title/Abstract])) OR (“insulin resistance”[Title/Abstract])) OR (“blood glucose”[Title/Abstract])) OR ("hyperglycemia"[Title/Abstract])) OR (hyperglycaemia[Title/Abstract]) OR (insulin[Title/Abstract])) OR (hyperinsulinemia*[Title/Abstract])) OR (hyperinsulinaemia*[Title/Abstract])) OR (bodyweight[Title/Abstract])) OR (“waist circumference”Title/Abstract]))
OR Fertility	((("fertility"[MeSH Terms]) OR ("pregnancy rate"[MeSH Terms])) OR (Fertility[Title/Abstract])) OR ("pre pregnancy"[Title/Abstract])

OR Fetal development	**(((("fetal blood"[MeSH Terms]) OR ("pregnancy complications"[MeSH Terms])) OR ("pregnancy outcome"[MeSH Terms])) OR ("gestational weight gain"[MeSH Terms])) OR ("fetal weight"[MeSH Terms])**
OR Bone health and bone density	((((((("bone density"[MeSH Terms]) OR ("bone and bones"[MeSH Terms])) OR (bone diseases, metabolic[MeSH Terms])) OR ("bone density"[Title/Abstract])) OR ("fractures, bone"[MeSH Terms])) OR (fracture*[Title/Abstract])) OR (osteoporo*[Title/Abstract])) OR (osteopeni*[Title/Abstract])) **AND** (((((("menopause"[MeSH Terms]) OR ("menopause"[MeSH Terms])) OR (postmenopausal[Title/Abstract])) OR (menopaus*[Title/Abstract])) OR (postmenopaus*[Title/Abstract])) OR (post-menopaus*[Title/Abstract]))
OR Infectious immunity	((((("immunity"[MeSH Terms]) OR ("infections"[MeSH Terms]) OR (immunity[Title/Abstract])) OR (immune[Title/Abstract])) OR (infection*[Title/Abstract])) AND (((((((((("aged"[MeSH Terms])) OR ("aged, 80 and over"[MeSH Terms])) OR (elderly[Title/Abstract])) OR (old[Title/Abstract])) OR (older[Title/Abstract])) OR (geriatric[Title/Abstract])) OR (pregnancy*[Title/Abstract])) OR (pregnant*[Title/Abstract])) OR (lactation*[Title/Abstract])) OR (lactating*[Title/Abstract]))
OR Independence	(((((((((((((((((((((((((((((((((("frailty"[MeSH Terms]) OR ("malnutrition"[MeSH Terms])) OR ("sarcopenia"[MeSH Terms])) OR (frailty[Title/Abstract])) OR (malnutrition[Title/Abstract])) OR (sarcopenic[Title/Abstract])) OR (sarcopenia[Title/Abstract])) OR ("muscle wasting"[Title/Abstract])) OR ("muscle maintenance"[Title/Abstract])) OR (ADLs[Title/Abstract])) OR (ADL[Title/Abstract])) OR ("physical function"[Title/Abstract])) OR (independence[Title/Abstract])) OR (falls[Title/Abstract])) OR (independent living[MeSH Terms])) OR ("functional status"[MeSH Terms])) OR ("accidental falls"[MeSH Terms])) OR OR ("fractures, bone"[MeSH Terms])) OR (fracture*[Title/Abstract])) OR (osteoporo*[Title/Abstract])) OR (osteopeni*[Title/Abstract])) OR ("activities of daily living"[MeSH Terms])) OR ("institutionalization"[MeSH Terms])) OR ("physical and rehabilitation medicine"[MeSH Terms]) OR ("osteoarthritis"[MeSH Terms]) OR ("arthritis, degenerative"[MeSH Terms])) OR ("degenerative arthritis"[MeSH Terms])) OR (arthritis[Title/Abstract])) OR (osteoarthritis*[Title/Abstract])) OR ("dementia"[MeSH Terms]) OR ("cognition"[MeSH Terms])) OR (dementia[Title/Abstract])) OR (alzheimer*[Title/Abstract])) OR (cognition[Title/Abstract])) OR (cognitive[Title/Abstract]) OR (Parkinson*[Title/Abstract]) OR (neurodegenerati*[Title/Abstract])<br>**AND**<br>((((("aged"[MeSH Terms]) OR ("aged, 80 and over"[MeSH Terms])) OR (elderly[Title/Abstract])) OR (old[Title/Abstract])) OR (older[Title/Abstract])) OR (geriatric[Title/Abstract])

Supplementary Table S2. Dietary intake data [1] detailing inadequately consumed nutrients per each demographic group.

Demographic Group	Inadequate Nutrient [1,2]	EAR[3]	AI [3]	% < EAR or AI [1]	Mean Intake [1,2]
**Children 4-8 years**	Dietary fiber		25 g/day	>97%	13.2-13.8 g/day
	Vitamin D	10 µg/day		>96%	4.7-5.5 µg/day
	Vitamin E	6 mg/day		33%-51%	6.2-7.2 mg/day
	Vitamin K		55 µg/day	53%-55%	56.5- 57.5 µg/day
	Choline		250 mg/day	60%-70%	221- 241 mg/day
	Calcium	800 mg/day		22%-37%	911-1040 mg/day
	Iodine	65 µg/day		>20%	Median intake 79-86 µg/day
	Potassium		2300 mg/day	69%-80%	1920-2082 mg/day
**Adolescent males 9-18 years**	Dietary fiber		31-38 g/day	>97%	15 - 15.3 g/day
	Vitamin A	445-630 µg/day		28-65%	571 - 622 µg/day
	Vitamin C	39-63 mg/day		20-62%	60.7 - 71.4 mg/day
	Vitamin D	10 µg/day		92-95%	4.7-5.4 µg/day
	Vitamin E	9-12 mg/day		61-83%	8.6 - 9 mg/day
	Choline		375-550 mg/day	92->97%	258 - 308 mg/day
	Calcium	1100 mg/day		56-62%	1036 - 1081 mg/day
	Iodine	73-95 ug/day		>40%	117 µg/day (median intake)
	Magnesium	200-340 mg/day		27-81%	247 - 276 mg/day
	Phosphorus	1055 mg/day		13-25%	1331 - 1505 mg/day
	Potassium		2500-3000 mg/day	75-84%	2147-2389 mg/day
**Adolescent females 9-18 years**	Protein	0.71-0.76 g/kgbw/day		3-23%	1.61-2.02 g/kgbw/day
	Dietary fiber		26 g/day	>97%	13.2-15.4 mg/day
	Vitamin A	420-485 µg/day		26-59%	474-605 µg/day
	Vitamin C	39-56 mg/day		16-59%	55.9-78.4 mg/day
	Vitamin D	10 µg/day		95 - >97%	3.2-4.8 µg/day
	Vitamin E	9-12 mg/day		60-94%	7.3-8.7 mg/day
	Vitamin K		60-75 µg/day	28-56%	75.9-82.1 µg/day

	Vitamin B6	0.8-1 mg/day		3-21%	1.46-1.77 mg/day
	Folate	250-330 μg/day		5-34%	415-517 μg/day
	Vitamin B12	1.5-2 ug/day		3-20%	3.34-4.33 μg/day
	Choline		375-400 mg/day	93->97%	214-252 mg/day
	Calcium	1100 mg/day		68-86%	811-987 mg/day
	Copper	540-685 μg/day		4-23%	900-1000 μg/day
	Iodine	73-95 μg/day		>40%	Median intake 108 μg/day
	Iron	5.7-7.9 mg/day		3-23%	11.6-14.5 mg/day
	Magnesium	200-300 mg/day		28-89%	218-246 mg/day
	Phosphorus	1055 mg/day		28-53%	1064-1291 mg/day
	Zinc	7-7.3 mg/day		20-49%	7.7-9.6 mg/day
	Potassium		2300 mg/day	64-83%	1818-2139 mg/day
**Adult males 19-70 years**	Dietary fiber		30-38 g/day	>97%	16.1-18.8 g/day
	Vitamin A	625 μg/day		51-65%	566-666 μg/day
	Vitamin C	75 mg/day		50-65%	68.7-87.5 mg/day
	Vitamin D	10 μg/day		93-97%	4.2-5.2 μg/day
	Vitamin E	12 mg/day		67-79%	9.3-10.8 mg/day
	Vitamin K		120 μg/day	50-73%	98.8-133.1 μg/day
	Choline		550 mg/day	88-92%	361-400 mg/day
	Calcium	800 mg/day		23-29%	1035-1097 mg/day
	Iodine	95 μg/day		>20%	Median intake 167 μg/day
	Magnesium	330-350 mg/day		53-64%	306-353 mg/day
	Zinc	9.4 mg/day		17-25%	12-13.2 mg/day
	Potassium		3400 mg/day	68-86%	2530-3028 mg/day
**Adult females 19-50 years**	Dietary fiber		25 g/day	94-95%	14.3-15.2 mg/day
	Vitamin A	500 μg/day		42-47%	557-587 μg/day
	Vitamin C	60 mg/day		48-52%	66.8-71.5 mg/day
	Vitamin D	10 μg/day		>97%	3.5-3.8 μg/day

	Vitamin E	12 mg/day		84%	8.7 mg/day
	Folate	320 µg/day		23-24%	431-432 µg/day
	Choline		425 mg/day	93-94%	282-286 mg/day
	Calcium	800 mg/day		42-46%	852-879 mg/day
	Iodine	95 µg/day		>20%	Median intake 161 µg/day
	Iron	8.1 mg/day		20-22%	11.9-12.1 mg/day
	Magnesium	255-265 mg/day		49-54%	256-276 mg/day
	Potassium		2600 mg/day	71-77%	2160-2287 mg/day
**Pregnancy and lactation 19-50 years**	Dietary fiber		28-29 g/day	mean intake < AI	17.3 g/day
	LA		13 g/day	mean intake < AI	11.94 g/day
	ALA		1.3-1.4 g/day	mean intake < AI	1.21 g/day
	Vitamin A	550-900 µg/day		28%	646-696 µg/day
	Vitamin C	70-100 mg/day		25%	80-122 mg/day
	Vitamin D	10 µg/day		92%	3.6-5.5 µg/day
	Vitamin E	12-16 mg/day		92%	6.3-7.8 mg/day
	Vitamin B6	1.6-1.7 mg/day		25%	1.8-2.1 mg/day
	Folate	450-520 µg/day		36%	492-630 µg/day
	Choline		450-550 mg/day	mean intake < AI	253-321 mg/day
	Calcium	800 mg/day		21%	888-1093 mg/day
	Iodine	160-209 µg/day		10-40%	181-216 µg/day
	Iron	6.5-22 mg/day		84%	14.7-17.2 mg/day
	Magnesium	265-300 mg/day		53%	254-294 mg/day
	Zinc	9.5-10.4 mg/day		22%	10.2-12.4 mg/day
	Potassium		2800 - 2900 mg/day	mean intake < AI	2346-2778 mg/day
**Menopause and post-menopause 51-70 years**	Dietary fiber		21 g/day	83%	15.5 g/day
	Vitamin A	500 µg/day		41%	592 µg/day
	Vitamin C	60 mg/day		45%	73.8 mg/day
	Vitamin D	10 µg/day		>97%	3.8 µg/day

	Vitamin E	12 mg/day		86%	8.4 mg/day
	Vitamin B6	1.3 mg/day		28%	1.67 mg/day
	Folate	320 μg/day		31%	400 μg/day
	Choline		425 mg/day	93%	292 mg/day
	Calcium	1000 mg/day		76%	832 mg/day
	Iodine	95 μg/day		>20%	Median intake 164 μg/day
	Magnesium	265 mg/day		54%	276 mg/day
	Potassium		2600 mg/day	65%	2397 mg/day
**Older adults >70 years**	Dietary fiber		21-30 g/day	84-92%	15.3-19.1 g/day
	Vitamin A	500-625 μg/day		27-37%	701-782 μg/day
	Vitamin C	60-75 mg/day		39-43%	80.6-96.3 mg/day
	Vitamin D	10 μg/day		89 - >97%	4.3-5.9 μg/day
	Vitamin E	12 mg/day		72-87%	8.2-10.2 mg/day
	Vitamin K		90-120 μg/day	35-55%	125.8-127.9 μg/day
	Vitamin B6	1.3-1.4 mg/day		19-28%	1.68-2.32 mg/day
	Folate	320 μg/day		13-29%	408-517 μg/day
	Choline		425-550 mg/day	90-94%	281-383 mg/day
	Calcium	1000 mg/day		58-82%	785-968 mg/day
	Iodine	95 μg/day		>20%	Median intake 158-163 μg/day
	Magnesium	265-350 mg/day		59-61%	256-330 mg/day
	Zinc	6.8-9.4 mg/day		26-29%	8.5-11.7 mg/day
	Potassium		2600-3400 mg/day	69%	2324-3026 mg/day

[1] Intake data were sourced from NHANES 2017 to prepandemic reports [1] for all demographic groups except pregnancy, where data were sourced from older NHANES intake reports [2,3] as well as supporting information from the scientific literature [4–8]. Table shows nutrients consumed at inadequate levels only; no excess nutrients were identified.
[2] Vitamin D values have been reported in μg/day to reflect survey method of reporting. 1 μg/day is equivalent to 40 IU/day.
[3] EAR and AI values as per the most recent DRI report (as applicable) [9–11].
AI, Adequate Intake; EAR, Estimated Average Requirement; DRI, Dietary Reference Intake; NHANES, National Health and Nutrition Examination Survey.

Supplementary Table S3. Summary of evidence[1] identified for nutrients where an increased need over the DRI has been identified per each demographic group.

Demographic Group	Nutrient	Mechanism	Suggested Revised Requirement
**Children 4-8 years**	Protein [12,13]	Updated IAAO data suggest current maintenance requirements, determined via nitrogen balance, are too low.	Up to 1.55 g/kgbw/day
	Magnesium [14]	Increased intake suggested to be necessary to retain a minimum level of magnesium for growth and adequate bone mineral content.	133 mg/day
**Adolescent males 9-18 years**	Zinc [15]	Suggested that data used to establish required zinc intakes may overestimate zinc absorption due to not accounting for the presence of phytates in the diet, leading to an underestimation of zinc requirements.	10% increase in requirements.
	Protein [12,13,16,17]	Updated IAAO data suggest current maintenance requirements, determined via nitrogen balance, are too low.	1.2 to 1.55 g/kgbw/day
	Magnesium [18]	Suggested update to magnesium requirement needed to account for factors that can affect the need for magnesium, such as calcium intake, obesity, deficiency in antioxidant nutrients, and a low protein diet.	Not provided.
**Adolescent females 9-18 years**	Protein [12,13,16,17]	Updated IAAO data suggest current maintenance requirements, determined via nitrogen balance, are too low.	1.2 to 1.55 g/kgbw/day
	Magnesium [18]	Suggested update to magnesium requirement needed to account for factors that can affect the need for magnesium, such as calcium intake, obesity, deficiency in antioxidant nutrients, and a low protein diet.	Not provided.
	Zinc [15]	Suggested that data used to establish required zinc intakes may overestimate zinc absorption due to not accounting for the presence of phytates in the diet, leading to an underestimation of zinc requirements.	10% increase in requirements.
**Adult males 19-70 years**	Vitamin C [19,20]	An increased dose was found to be necessary to achieve maximum bioavailability, and plasma and tissue concentrations. The daily vitamin C intake needed to prevent collagen-related pathologies is > 75 to 110 mg/day.	>75-200 mg/day
	Protein [13,16,17]	Updated IAAO data suggest current maintenance requirements, determined via nitrogen balance, are too low.	0.99-1.2 g/kgbw/day

	Magnesium [18]	Suggested update to magnesium requirement needed to account for factors that can affect the need for magnesium, such as calcium intake, obesity, deficiency in antioxidant nutrients, and a low protein diet.	Not provided.
	Zinc [15]	Suggested that data used to establish required zinc intakes may overestimate zinc absorption due to not accounting for the presence of phytates in the diet, leading to an underestimation of zinc requirements.	13% increase in requirements.
**Adult females 19-50 years**	Protein [13,16,17]	Updated IAAO data suggest current maintenance requirements, determined via nitrogen balance, are too low.	0.99-1.2 g/kgbw/day
	Vitamin C [19,20]	An increased dose was found to be necessary to achieve maximum bioavailability, and plasma and tissue concentrations. The daily vitamin C intake needed to prevent collagen-related pathologies is > 75 to 110 mg/day.	>75-200 mg/day
	Magnesium [18]	Suggested update to magnesium requirement needed to account for factors that can affect the need for magnesium, such as calcium intake, obesity, deficiency in antioxidant nutrients, and a low protein diet.	Not provided.
	Zinc [15]	Suggested that data used to establish required zinc intakes may overestimate zinc absorption due to not accounting for the presence of phytates in the diet, leading to an underestimation of zinc requirements.	15% increase in requirements.
**Pregnancy and lactation 19-50 years**	Protein [13,16,17]	Updated IAAO data suggest current maintenance requirements, determined via nitrogen balance, are too low. It is likely that further increases will be needed for pregnancy and lactation.	At least 0.99-1.2 g/kgbw/day
	Zinc [15]	Suggested that data used to establish required zinc intakes may overestimate zinc absorption due to not accounting for the presence of phytates in the diet, leading to an underestimation of zinc requirements.	15% increase in requirements.
	Magnesium [18]	Suggested update to magnesium requirement needed to account for factors that can affect the need for magnesium, such as calcium intake, obesity, deficiency in antioxidant nutrients, and a low protein diet.	Not provided.
**Menopause and post-menopause 51-70 years**	Vitamin C [19,20]	An increased dose was found to be necessary to achieve maximum bioavailability, and plasma and tissue concentrations.	200 mg/day
	Vitamin D [21]	Decreased absorption during and post-menopause.	Not provided.

	Protein [13,16,17]	Updated IAAO data suggest current maintenance requirements, determined via nitrogen balance, are too low. It is likely that further increases will be needed for pregnancy and lactation.	At least 0.99-1.2 g/kgbw/day
	Magnesium [18]	Suggested update to magnesium requirement needed to account for factors that can affect the need for magnesium, such as calcium intake, obesity, deficiency in antioxidant nutrients, and a low protein diet.	Not provided.
	Zinc [15]	Suggested that data used to establish required zinc intakes may overestimate zinc absorption due to not accounting for the presence of phytates in the diet, leading to an underestimation of zinc requirements.	15% increase in requirements.
**Older adults >70 years**	Protein [13,22]	Older adults have higher requirements to achieve homeostasis due to anabolic resistance. Experts in the field of protein and aging have recommended that the optimal protein intake for elderly adults.	1.2-2 g/kgbw/day
	Vitamin B12 [23]	Recalculation of the RDA for vitamin B12 in older women.	17% increase in RDA.
	Vitamin C [19,20]	An increased dose was found to be necessary to achieve maximum bioavailability, and plasma and tissue concentrations. Recalculation of the RDA for vitamin C in older women.	200 mg/day
	Vitamin D [21,23]	There is higher variability in the vitamin D need in this group due to physiological changes among individuals, including reduced efficiency of vitamin D synthesis in skin and reduced endogenous production of active vitamin D; this is reflected in the RDA for vitamin D for adults over 70 years, but not the EAR. Increased dietary intake is required to maintain homeostasis in older adults and to maintain bone health. Decreased absorption is known to occur during ageing.	Not provided.
	Magnesium [18]	Suggested update to magnesium requirement needed to account for factors that can affect the need for magnesium, such as calcium intake, obesity, deficiency in antioxidant nutrients, and a low protein diet.	Not provided.
	Zinc [24]	Increased risk of zinc malabsorption due to highly prevalent malabsorptive factors during older age, including muscle wasting and polypharmacy.	Not provided.

	Zinc [15]	Suggested that data used to establish required zinc intakes may overestimate zinc absorption due to not accounting for the presence of phytates in the diet, leading to an underestimation of zinc requirements.	13-15% increase in requirements.

[1] Where consensus was identified in the scientific literature. No evidence for decreased needs, relative to the DRI, was found.

EAR, Estimated Average Requirement; DRI, Dietary Reference Intake; IAAO, indicator amino acid oxidation; RDA, Recommended Dietary Allowance.

Supplementary Table S4. Summary of evidence[1] identified for nutrients showing an association with one or more health priorities per each demographic group.

Demographic Group	Nutrient	Priority Health Outcome	Effect	Direction of effect	Dose range associated with effect[2]
**Children 4-8 years**	Calcium	Growth and development	↑ BMD, BMC [25,26]	Beneficial	460-850 mg/day
	Iron	Growth and development	↑ cognitive performance [27]	Beneficial	1.66-60 mg/day
	Zinc	Growth and development	↑ cognitive performance, weight, height [27,28]	Beneficial	1.66-5.6 mg/day
	Vitamin B12	Growth and development	↑ cognitive performance [27]; ↓ B12 status in ASD and ADHD subjects [29]	Beneficial	1.04-1.17 μg/day
	Vitamin D	Growth and development	↑ BMD, BMC [30–32], cognitive function [33]; ↓ hyperactivity scores in ASD subjects [34]	Beneficial	132-2000 IU/day
		Respiratory health	↓ allergic rhinitis symptoms [35], respiratory tract infections [36]	Beneficial	800-1000 IU/day
		Metabolic health	↓ HOMA-IR, BMI [37,38]	Beneficial	600->4000 IU/day
		Mood and mental health	↑ mood, wellbeing [39]	Beneficial	1000 IU/day – 50000 IU/week
	Omega 3	Growth and development	↑ learning, memory, decision making [40,41]	Beneficial	≥0.45 g/day long chain omega 3
		Mood and mental health	↓ depression as adjunct therapy [42]	Beneficial	Not provided
	Folate	Growth and development	↑ folate status in ADHD subjects [29]	Adverse	H vs L blood levels
**Adolescent males 9-18 years**	Calcium	Growth and development	↑ BMD, BMC [25,26]	Beneficial	460 to 850 mg /day
	Vitamin D	Growth and development	↑ BMD, BMC [30–32], cognitive function [33]	Beneficial	132-2000 IU/day
		Respiratory health	↓ allergic rhinitis symptoms [35], respiratory tract infections [36], asthma attacks [43,44]	Beneficial	500-4000 IU/day
		Mood and mental health	↑ mood, wellbeing [39]	Beneficial	1000 IU/day-50000 IU/week
		Metabolic health	↓ HOMA-IR, BMI [37,38]	Beneficial	600->4000 IU/day

		Cancer	↓ risk renal cell carcinoma, risk liver cancer, risk breast cancer, melanoma thickness [45–48]	Beneficial	H vs L blood status
	Iron	Growth and development	↑ cognitive performance [27]	Beneficial	1.66-60 mg/day
	Zinc	Growth and development	↑ cognitive performance, weight, height [27,28]	Beneficial	1.66-5.6 mg/day
	Vitamin B12	Growth and development	↑ cognitive performance [27]; ↓ B12 status in ASD and ADHD subjects [29]	Beneficial	1.04-1.17 µg/day
	Omega 3	Growth and development	↑ learning, memory, decision making [41]	Beneficial	≥0.45 g/day long chain omega 3s
		Mood and mental health	↓ depression as adjunct therapy [42]	Beneficial	Not provided
	Folate	Growth and development	↑ folate status in ADHD subjects [29]	Adverse	H vs L blood levels
**Adolescent females 9-18 years**	Calcium	Growth and development	↑ BMD, BMC [25,26]	Beneficial	460 to 850 mg/day
	Vitamin D	Growth and development	↑ BMD, BMC [30–32], cognitive function [33]	Beneficial	132-2000 IU/day
		Respiratory health	↓ allergic rhinitis symptoms [35], respiratory tract infections [36], asthma attacks [43,44]	Beneficial	500-4000 IU/day
		Mood and mental health	↑ mood, wellbeing [39]	Beneficial	1000 IU/day - 50000 IU/week
		Metabolic health	↓ HOMA-IR, BMI [37,38]	Beneficial	600->4000 IU/day
		Cancer	↓ risk renal cell carcinoma, risk liver cancer, risk breast cancer, melanoma thickness [45–48]	Beneficial	H vs L blood status
	Iron	Growth and development	↑ cognitive performance [27]	Beneficial	1.66-60 mg/day
	Zinc	Growth and development	↑ cognitive performance, weight, height [27,28]	Beneficial	1.66-5.6 mg/day
	Vitamin B12	Growth and development	↑ cognitive performance [27]; ↓ B12 status in ASD and ADHD subjects [29]	Beneficial	1.04-1.17 µg/day
	Protein	Growth and development	↑ risk for early menarche onset [49]	Adverse	H vs L intake
	Dietary fiber	Growth and development	↓ risk for early menarche onset [49]	Beneficial	H vs L intake
	Omega 3	Growth and development	↑ learning, memory, decision making [41]	Beneficial	≥0.45 g/day long chain omega 3s
		Mood and mental health	↓ depression as adjunct therapy [42]	Beneficial	Not provided
	Folate	Growth and development	↑ folate status in ADHD subjects [29]	Adverse	H vs L blood levels
**Adult males 19-70 years**	Vitamin D	Mood and mental health	↑ mental health, quality of life [39,50]; ↓ depression, anxiety [39,51–54]	Beneficial	≥2000-14000 IU/day (equivalent)
		Metabolic health	↓ WHR, FBG, FBI, HOMA-IR, BMI, WC, risk of T2DM [36,55–57]; ↑ QUICKI [57]	Beneficial	1000-7142 IU/day (equivalent); H vs L blood levels

	Cancer	↓ risk renal cell carcinoma, risk liver cancer, risk breast cancer, risk lung cancer, risk colorectal cancer, risk ovarian cancer, risk cancer mortality, melanoma thickness [45–48,58–60]; ↑ survival after diagnosis [61]	Beneficial	1200-8000 IU/day, H vs L blood levels, H vs L intake
	Cardiovascular health	↓ risk CVD events, risk CVD mortality, risk stroke BP, CRP [57,62–71]	Beneficial	S vs I/D blood status, H vs L blood status, 1000-7100 IU/day equivalent
Omega 3	Mood and mental health	↓ depression symptoms or severity, psychological distress, anxiety [72–76]	Beneficial	1-6.6 g/day total omega-3s
	Metabolic health	↓ risk for T2DM [77]	Beneficial	H vs L blood levels
	Cancer	↓ risk for colorectal cancer [77,78]; ↑ cancer survival [79]	Beneficial	H vs L blood levels; H vs L intake
	Cardiovascular health	↓ risk CVD, risk CHD, risk stroke, risk MI, risk CVD-related mortality, TG, TC, plasma Hcy, non-HDL-C, CRP, SBP [63,77,80–90]; ↑ FMD [91,92]	Beneficial	H vs L blood levels; 0.34-4 g/day
	Cardiovascular health	↑ risk AF [89]	Adverse	0.28-5.4 g/day
Vitamin C	Mood and mental health	↑ mood [93]	Beneficial	100-3000 mg/day
	Cardiovascular health	↑ endothelial function [94]; ↓ risk CVD mortality [95]	Beneficial	500-2000 mg/day; H vs L intake; H vs L serum levels
Vitamin E	Cardiovascular health	↓ fatal MI, risk CVD mortality, SBP [96,97]; ↑ endothelial function [97]	Beneficial	54-1200 mg/day; H vs L serum levels
	Cancer	↓ risk bladder cancer, risk esophagal cancer, risk lung cancer, risk kidney cancer, risk pancreatic cancer [97]	Beneficial	10 mg/day; H vs L intake; H vs L serum levels
Folate	Mood and mental health	↓ depression symptoms as adjunct therapy [98,99]	Beneficial	0.5 to 10 mg/day folic acid; 7.5 to 15 mg/day 5-MTHF
	Metabolic health	↓ FBG, FBI, HOMA-IR [100]	Beneficial	0.8-5 mg/day folic acid; 15 mg/day 5-MTHF
	Cancer	↓ risk for colorectal cancer, pathological changes associated with gastric precancerous conditions [101,102]	Beneficial	H vs L intake, 20-30 mg/day folic acid

		Cardiovascular health	↓ risk stroke, risk CVD, carotid intimid-media thickness [63,103]	Beneficial	H vs L folate; folic acid intake
	Zinc	Mood and mental health	↓ depression symptoms, risk of depression [104,105]	Beneficial	7-25 mg/day; H vs L blood levels
	Dietary fiber	Metabolic health	↓ risk for T2DM, fasting plasma glucose, fasting plasma insulin, HOMA-IR, HbA1c, BW, BMI [106–108]	Beneficial	3-30 g/day; H vs L intake
		Cancer	↓ risk colon cancer, risk pancreatic cancer, risk gastric cancer, risk liver cancer, risk cancer-related mortality [109–113]	Beneficial	10 g/day, H vs L intake
		Cardiovascular health	↓ risk CVD, risk CHD, risk CVD mortality, risk CAD, risk stroke, TC, LDL-C, SBP, DBP [106,108,110–112,114,115]	Beneficial	H vs L intake; DR per each 10-15 g/day; 3-35 g/day
	Calcium	Metabolic health	↓ BMI [116]	Beneficial	≥ 1000 mg/day
	Omega 6	Cancer	↓ risk prostate cancer in males [117]	Beneficial	H vs L tissue levels
		Cardiovascular health	↓ risk CVD, risk CVD mortality, risk stroke, risk fatal CHD [87,118,119]	Beneficial	H vs L intake (LA); H vs L blood levels (LA)
	Protein	Cardiovascular health	↓ risk CVD [120]	Beneficial	H vs L intake
	Vitamin B12	Cardiovascular health	↓ risk CVD, Hcy [103,121]	Beneficial	H vs L intake; >500 µg/day
	Copper	Cardiovascular health	↑ risk stroke, risk CAD mortality, risk CVD mortality [122]	Adverse	H vs L blood levels
	Vitamin B6	Cardiovascular health	↓ risk CVD [103]	Beneficial	H vs L intake
**Adult females 19-50 years**	Vitamin D	Mood and mental health	↑ mental health, quality of life [39,50]; ↓ depression, anxiety [39,51–54]	Beneficial	≥2000-14000 IU/day (equivalent)
		Metabolic health	↓ WHR, FBG, FBI, HOMA-IR, BMI, WC [36,56,57]; ↑ QUICKI [57]	Beneficial	1000-7142 IU/day (equivalent)
		Cancer	↓ risk renal cell carcinoma, risk liver cancer, risk breast cancer, risk lung cancer, risk colorectal cancer, risk ovarian cancer, risk cancer mortality, melanoma thickness [45–48,58–60]	Beneficial	>2000 IU/day, H vs L blood levels, H vs L intake
		Cardiovascular health	↓ risk CVD events, risk CVD mortality, risk stroke BP, CRP [57,62,64,65,67–71]	Beneficial	S vs I/D blood status, H vs L blood status; 1000-7100 IU/day equivalent

	Fertility	↑ AMH, IVF outcomes, clinical pregnancy rate [123–125]	Beneficial	S vs I/D blood status; 1000-7100 IU/day equivalent
Omega 3	Mood and mental health	↓ depression symptoms or severity, psychological distress, anxiety [72–76]	Beneficial	1000-6600 mg/day
	Metabolic health	↓ risk for T2DM [77]	Beneficial	H vs L blood levels
	Cancer	↓ risk for colorectal cancer [77,78]; ↑ cancer survival [79]	Beneficial	H vs L blood levels; H vs L intake
	Cardiovascular health	↓ risk CVD, risk CHD, risk stroke, risk CVD-related mortality, TG, TC, plasma Hcy, non-HDL-C, CRP, SBP [63,77,80,81,83,84,86,88–90]; ↑ FMD [91,92]	Beneficial	H vs L blood levels; 0.4-3.8 g/day
	Cardiovascular health	↑ risk AF [89]	Adverse	0.28-5.4 g/day
	Fertility	↑ IVF rate [126]	Beneficial	1-2 g/day; H vs L intake
Vitamin C	Mood and mental health	↑ mood [93]	Beneficial	100-3000 mg/day
	Cardiovascular health	↑ endothelial function [94]; ↓ risk CVD mortality [95]	Beneficial	500-2000 mg/day; H vs L intake; H vs L serum levels
Vitamin E	Cardiovascular health	↓ fatal MI, risk CVD mortality, SBP [96,97]; ↑ endothelial function [97]	Beneficial	54-1200 mg/day; H vs L serum levels
	Cancer	↓ risk bladder cancer, risk esophagal cancer, risk lung cancer, risk kidney cancer, risk pancreatic cancer [97]	Beneficial	10 mg/day; H vs L intake; H vs L serum levels
Folate	Mood and mental health	↓ depression symptoms as adjunct therapy [98,99]	Beneficial	0.5 to 10 mg/day folic acid; 7.5 to 15 mg/day L-Methylfolate
	Metabolic health	↓ FBG, FBI, HOMA-IR [100]	Beneficial	0.8-5 mg/day folic acid; 15 mg/day 5-MTHF
	Cancer	↓ risk for colorectal cancer, risk for breast cancer, pathological changes associated with gastric precancerous conditions [101,102,127]	Beneficial	H vs L intake, 20-30 mg/day folic acid
	Cardiovascular health	↓ risk CVD, carotid intimid-media thickness [103]	Beneficial	H vs L folate intake
Zinc	Mood and mental health	↓ depression symptoms, risk of depression [104,105]	Beneficial	7-25 mg/day; H vs L blood levels

	Dietary fiber	Metabolic health	↓ risk for T2DM, fasting plasma glucose, fasting plasma insulin, HOMA-IR, HbA1c, BW, BMI [106–108]	Beneficial	3-30 g/day; H vs L intake
		Cancer	↓ risk colon cancer, risk pancreatic cancer, risk gastric cancer, risk liver cancer, risk breast cancer, risk cancer-related mortality [109–113,128]	Beneficial	10 g/day, H vs L intake
		Cardiovascular health	↓ risk CVD, risk CHD, risk CVD mortality, risk CAD, risk stroke, TC, LDL-C, SBP, DBP [106,108,110–112,114,115]	Beneficial	H vs L intake; DR per each 10-15 g/day; 3-35 g/day
	Calcium	Metabolic health	↓ BMI [116]	Beneficial	≥ 1000 mg/day
		Cancer	↓ risk breast cancer [129]	Beneficial	350 mg/day
	Omega 6	Cardiovascular health	↓ risk fatal CHD [119]	Beneficial	H vs L blood levels (LA)
	Protein	Cardiovascular health	↓ risk CVD [120]	Beneficial	H vs L intake
	Vitamin B12	Cardiovascular health	↓ risk CVD, Hcy [103,121]	Beneficial	H vs L intake; >500 µg/day
	Copper	Cardiovascular health	↑ risk stroke, risk CAD mortality, risk CVD mortality [122]	Adverse	H vs L blood levels
	Vitamin B6	Cardiovascular health	↓ risk CVD [103]	Beneficial	H vs L intake
**Pregnancy and lactation 19-50 years**	Vitamin D	Mood and mental health	↓ risk depression [130,131]	Beneficial	S vs D blood status
		Metabolic health	↓ risk GDM, HOMA-IR, FBG, FBI [132–137]; ↑ glucose tolerance [132]	Beneficial	400-3570 IU/day; S vs D blood status
		Cardiovascular health	↓ risk preeclampsia, LDL-C [132,133,135,138–140]	Beneficial	> 400 IU/day
		Fetal development	↓ risk premature birth, risk VDD, risk asthma, risk wheeze, risk dental caries in childhood, risk SGA, risk congenital abnormality, hyperbilirubinemia, neonatal hospitalization, wheezing [36,133,136,140–144]; ↑ BMD, birth length, bone growth, live birth rate [123,135,145–148]	Beneficial	S vs D blood status; H vs L blood status; >400-7100 IU/day equivalent
	Vitamin E	Fetal development	↓ risk asthmatic disease, risk wheeze in children [149]	Beneficial	7.9-400 mg/day
	Omega 3	Mood and mental health	↓ depression symptoms [150]	Beneficial	0.3-1 g/day
		Metabolic health	↓ fasting glucose, postprandial glucose, FBI, HOMA-IR in pregnant women with GDM [151]	Beneficial	1-2 g/day

		Cardiovascular health	↓ risk preeclampsia, TG, LDL-C, CRP [152–154]; ↑ HDL-C [153]	Beneficial	0.2-3 g/day
		Fetal development	↓ low birth weight, premature birth [154,155]	Beneficial	0.3-3 g/day
	Dietary fiber	Metabolic health	↓ fasting glucose, postprandial glucose, and HbA1c in pregnant women with GDM [151]	Beneficial	12 g/day
		Cardiovascular health	↓ TC, LDL-C in pregnant women with GDM [151]	Beneficial	12 g/day
		Fetal development	↓ premature birth, caesarean delivery, fetal distress for pregnant women with GDM [151]	Beneficial	12 g/day
	Folate	Metabolic health	↑ GDM risk [156]	Adverse	H vs L blood levels; prolonged supplementation
		Fetal development	↓ risk birth defects, risk neurodevelopmental issues, risk respiratory allergic diseases [157–159]	Beneficial	0.4-0.8 mg/day
	Magnesium	Metabolic health	↓ FBG, FBI, HOMA-IR in women with GDM [160]; ↑ QUICKI [160]	Beneficial	250 mg/day MgO; 150 mg Mq equivalent
		Cardiovascular health	↓ TC, LDL-C [160]	Beneficial	250 mg/day MgO; 150 mg Mq equivalent
	Zinc	Metabolic health	↓ FBG, FBI, HOMA-IR in women with GDM [161]	Beneficial	8 to 30 mg/day
		Infectious immunity	↓ CRP during infection [162]	Beneficial	12-30 mg/day
	Calcium	Cardiovascular health	↓ risk preeclampsia, risk HT [139,163–166]	Beneficial	120-2000 mg/day
	Iodine	Fetal development	↓ risk SGA [167]; ↑ psychomotor development [168]	Beneficial	H vs L iodine status; up to 300 μg/day
**Menopause and post-menopause 51-70 years**	Vitamin D	Mood and mental health	↑ mental health, quality of life [39,50]; ↓ depression, anxiety [39,51–54]	Beneficial	≥2000-14000 IU/day (equivalent)
		Metabolic health	↓ WHR, FBG, FBI, HOMA-IR, BMI, WC, risk of T2DM [36,55–57]; ↑ QUICKI [57]	Beneficial	1000-7142 IU/day (equivalent); H vs L blood levels
		Cancer	↓ risk renal cell carcinoma, risk liver cancer, risk breast cancer, risk lung cancer, risk colorectal cancer, risk ovarian cancer, risk cancer mortality, melanoma thickness [45–48,58–60]; ↑ survival after diagnosis [61]	Beneficial	1200-8000 IU/day, H vs L blood levels, H vs L intake

	Cardiovascular health	↓ risk CVD events, risk CVD mortality, risk stroke BP, CRP [57,62–71]	Beneficial	S vs I/D blood status, H vs L blood status; 1000-7100 IU/day equivalent
	Bone health and density	↓ risk osteoporosis, risk fracture [169,170]; ↑ BMD, bone remodelling markers [171,172]	Beneficial	H vs L blood levels; 400-1000 IU/day
Omega 3	Mood and mental health	↓ depression symptoms or severity, psychological distress, anxiety [72–76,173,174]	Beneficial	0.5-6.6 g/day; H vs L intake
	Metabolic health	↓ risk for T2DM [77]	Beneficial	H vs L blood levels
	Cancer	↓ risk for colorectal cancer [77,78]; ↑ cancer survival [79]	Beneficial	H vs L blood levels; H vs L intake
	Cardiovascular health	↓ risk CVD, risk CHD, risk stroke, risk MI, risk CVD-related mortality, TG, TC, plasma HC, non-HDL-C, CRP, SBP [63,77,80–90]; ↑ FMD [91,92]	Beneficial	H vs L blood levels; 0.34-4 g/day
	Cardiovascular health	↑ risk AF [89]	Adverse	0.28-5.4 g/day
Vitamin C	Mood and mental health	↑ mood [93]	Beneficial	100-3000 mg/day
	Cardiovascular health	↑ endothelial function [94]; ↓ risk CVD mortality [95]	Beneficial	500-2000 mg/day; H vs L intake; H vs L blood levels
Vitamin E	Cardiovascular health	↓ fatal MI, risk CVD mortality, SBP [96,97]; ↑ endothelial function [97]	Beneficial	54-1200 mg/day; H vs L blood levels
	Cancer	↓ risk bladder cancer, risk esophagal cancer, risk lung cancer, risk kidney cancer, risk pancreatic cancer [97]	Beneficial	10 mg/day; H vs L intake; H vs L blood levels
Folate	Mood and mental health	↓ depression symptoms as adjunct therapy [98,99]	Beneficial	0.5 to 10 mg/day folic acid; 7.5 to 15 mg/day L-Methylfolate
	Metabolic health	↓ FBG, FBI, HOMA-IR [100]	Beneficial	0.8-5 mg/day folic acid; 15 mg/day 5-MTHF
	Cancer	↓ risk for colorectal cancer, pathological changes associated with gastric precancerous conditions [101,102]	Beneficial	H vs L intake, 20-30 mg/day folic acid
	Cardiovascular health	↓ risk stroke, risk CVD, carotid intimid-media thickness [63,103]	Beneficial	H vs L folate; folic acid intake
Zinc	Mood and mental health	↓ depression symptoms, risk of depression [104,105]	Beneficial	7-25 mg/day; H vs L blood levels

	Dietary fiber	Metabolic health	↓ risk for T2DM, fasting plasma glucose, fasting plasma insulin, HOMA-IR, HbA1c, BW, BMI [106–108]	Beneficial	3-30 g/day; H vs L intake
		Cancer	↓ risk colon cancer, risk pancreatic cancer, risk gastric cancer, risk liver cancer, risk breast cancer, risk cancer-related mortality [109–113,128]	Beneficial	10 g/day, H vs L intake
		Cardiovascular health	↓ risk CVD, risk CHD, risk CVD mortality, risk CAD, risk stroke, TC, LDL-C, SBP, DBP [106,108,110–112,114,115]	Beneficial	H vs L intake; DR per each 10-15 g/day; 3-35 g/day
	Calcium	Metabolic health	↓ BMI [116]	Beneficial	≥ 1000 mg/day
		Cancer	↓ risk breast cancer [129]	Beneficial	350 mg/day
		Bone health and density	↑ BMD [175,176]	Beneficial	1200 mg/day
	Omega 6	Cardiovascular health	↓ risk CVD, risk CVD mortality, risk stroke, risk fatal CHD [87,118,119]	Beneficial	H vs L intake (LA); H vs L blood levels (LA)
	Protein	Cardiovascular health	↓ risk CVD [120]	Beneficial	H vs L intake
		Bone health and density	↑ lumbar spine BMD [177]	Beneficial	10-90 g/day
	Vitamin B12	Cardiovascular health	↓ risk CVD, Hcy [103,121]	Beneficial	H vs L intake; >500 μg/day
	Copper	Cardiovascular health	↑ risk stroke, risk CAD mortality, risk CVD mortality [122]	Adverse	H vs L blood levels
	Vitamin B6	Cardiovascular health	↓ risk CVD [103]	Beneficial	H vs L intake
**Older adults >70 years**	Dietary fiber	Metabolic health	↓ risk for T2DM, fasting plasma glucose, fasting plasma insulin, HOMA-IR, HbA1c, BW, BMI [106–108]	Beneficial	3-30 g/day; H vs L intake
		Cancer	↓ risk colon cancer, risk pancreatic cancer, risk gastric cancer, risk liver cancer, risk breast cancer, risk cancer-related mortality [109–113,128]	Beneficial	10 g/day, H vs L intake
		Cardiovascular health	↓ risk CVD, risk CHD, risk CVD mortality, risk CAD, risk stroke [106,110–112,114,115]	Beneficial	H vs L intake; DR per each 10 g/day; 3-30 g/day
	Omega 3	Metabolic health	↓ risk for T2DM [77]	Beneficial	H vs L blood levels
		Cancer	↓ risk for colorectal cancer [77,78]; ↑ cancer survival [79]	Beneficial	H vs L blood levels; H vs L intake

		Cardiovascular health	↓ risk CVD, risk CHD, risk stroke, risk MI, risk CVD-related mortality, TG, TC, plasma HC, non-HDL-C, CRP, SBP [63,77,80–90]; ↑ FMD [91,92]	Beneficial	H vs L blood levels; 0.34-4 g/day
		Cardiovascular health	↑ risk AF [89]	Adverse	0.28-5.4 g/day
		Independence	↑ lower body strength, sit to stand performance, up and go performance, physical function, memory, attention [178–187]; ↓ pain, risk cognitive decline [180,188]	Beneficial	H vs L intake; 0.23-5 g/day EPA+DHA; 1.35-14 g/day ALA
	Vitamin D	Metabolic health	↓ WHR, FBG, FBI, HOMA-IR, risk of T2DM [36,55,57]; ↑ QUICKI [57]	Beneficial	1000-7142 IU/day (equivalent); H vs L blood levels
		Cancer	↓ risk renal cell carcinoma, risk liver cancer, risk breast cancer, risk lung cancer, risk colorectal cancer, risk ovarian cancer, risk cancer mortality, melanoma thickness [45–48,58–60]; ↑ survival after diagnosis [61]	Beneficial	1200-8000 IU/day, H vs L blood levels, H vs L intake
		Cardiovascular health	↓ risk CVD events, risk CVD mortality, risk stroke BP, CRP [57,62–71]	Beneficial	S vs I/D blood status, H vs L blood status; 1000-7100 IU/day equivalent
		Infectious immunity	↓ risk severe COVID-19, risk COVID-19 mortality [189]	Beneficial	≥ 1000 IU/day
		Independence	↑ quadriceps strength, bone remodelling markers, cognitive function [172,190,191]; ↓ risk of falls, risk mortality after hip fracture, risk knee osteoarthritis, risk post-surgery cognitive dysfunction [191–196]	Beneficial	H vs L blood status; S vs D blood status; 400- <1000 IU/day; 0.25-1 ug/day prescription forms of vitamin D (active)
		Independence	↑ risk falls, hip fracture [195,197]; ↓ physical performance [198]	Adverse	Doses >1000 IU/day, large intermittent doses
	Vitamin E	Cardiovascular health	↓ fatal MI, risk CVD mortality [97]; ↑ endothelial function [97]	Beneficial	134-1200 mg/day; H vs L serum levels
		Cancer	↓ risk bladder cancer, risk esophagal cancer, risk lung cancer, risk kidney cancer, risk pancreatic cancer [97]	Beneficial	10 mg/day; H vs L intake; H vs L serum levels
	Vitamin C	Cardiovascular health	↑ endothelial function [94]; ↓ risk CVD mortality [95]	Beneficial	500-2000 mg/day; H vs L intake; H vs L serum levels

Calcium	Metabolic health	↓ BMI [116]	Beneficial	≥ 1000 mg/day
	Cancer	↓ risk breast cancer [129]	Beneficial	350 mg/day
Omega 6	Cancer	↓ risk prostate cancer in males [117]	Beneficial	H vs L tissue levels
	Cardiovascular health	↓ risk CVD, risk CVD mortality, risk stroke, risk fatal CHD [87,118,119]	Beneficial	H vs L intake (LA); H vs L blood levels (LA)
Folate	Cancer	↓ risk for colorectal cancer [102]	Beneficial	H vs L intake
	Cardiovascular health	↓ risk stroke, risk CVD, carotid intimid-media thickness [63,103]	Beneficial	H vs L folate; folic acid intake
	Independence	↑ global cognitive function, memory, attention [178,199,200]; ↓ risk cognitive decline, risk AD [201,202]	Beneficial	H vs L blood status; H vs L intake; 0.4-5 mg folic acid
Protein	Cardiovascular health	↓ risk CVD [120]	Beneficial	H vs L intake
	Independence [87,118,119]	↑ muscle protein synthesis, muscle mass, muscle performance, aerobic capacity, leg strength, increased lean body mass, muscle strength, hand-grip strength, walking capability, gait speed, sarcopenia outcomes [203–219]; ↓ risk sarcopenia, risk frailty, risk for falls, body fat, pain associated with osteoarthritis [204–207,209,210,214,220,221]	Beneficial	4-100 g/day; 1.1-1.8 g/kgbw/day; 6-15 g/day EAAs; 2.5-20 g/day BCAAs; 0.9-7.5 g/day leucine (often with resistance training)
Vitamin B12	Cardiovascular health	↓ risk CVD, Hcy [103,121]	Beneficial	H vs L intake; >500 µg/day
Copper	Cardiovascular health	↑ risk stroke, risk CAD mortality, risk CVD mortality [122]	Adverse	H vs L blood levels
Vitamin B6	Cardiovascular health	↓ risk CVD [103]	Beneficial	H vs L intake
Zinc	Infectious immunity	↓ CRP during infection [162]	Beneficial	15-100 mg/day
Magnesium	Independence	↓ risk dementia, risk cognitive impairment (U-shaped association) [222]	Beneficial	H vs L blood levels

[1] Based on consistent evidence from SLRs of RCTs and/or prospective cohort studies.

[2] Dose represents amount added to normal diet, unless otherwise stated. For vitamin D, 1 µg is equivalent to 40 IU. For protein, intake is reported as g/day or g/kgbw/day, depending on how data have been presented within the source literature.

SLR, systematic literature review; RCT, randomized controlled trial; H, high; L, low; S, sufficient; I, insufficient; D, deficient; IU, international units; ADHD, Attention Deficit Hyperactivity Disorder; ASD, Autism Spectrum Disorder; AMH, anti-müllerian hormone; AD, Alzheimer's disease; BMI, body mass index; BMC, bone mineral content; BMD, bone mineral density; COVID-19, Coronavirus disease 2019; CVD, cardiovascular disease; CAD, coronary artery disease; CHD, coronary heart disease; Hcy, homocysteine;

EAA, essential amino acid; BCAA, branched chain amino acid; 5-MTHF, 5 methyltetrahydrofolate; IVF, *in vitro* fertilization; SGA, small for gestational age; VDD, vitamin D deficiency; HOMA-IR, Homeostatic Model of Insulin Resistance; WHR, waist to hip ratio; FBG, fasting blood glucose; FBI, fasting blood insulin; WC, waist circumference; T2DM, type 2 diabetes mellitus; GDM, gestational diabetes mellitus; BW, body weight;HbA1c, glycated hemogloblin; QUICKI, quantitative insulin sensitivity check index; DBP, diastolic blood pressure; SBP, systolic blood pressure; TC, total cholesterol; LDL-C, low density lipoprotein cholesterol; HDL-C, high density lipoprotein cholesterol; BP, blood pressure, HT, hypertension; CRP, C-reactive protein; TG, triglycerides; MI, myocardial infarction; AF, atrial fibrillation; FMD, flow mediated dilation.

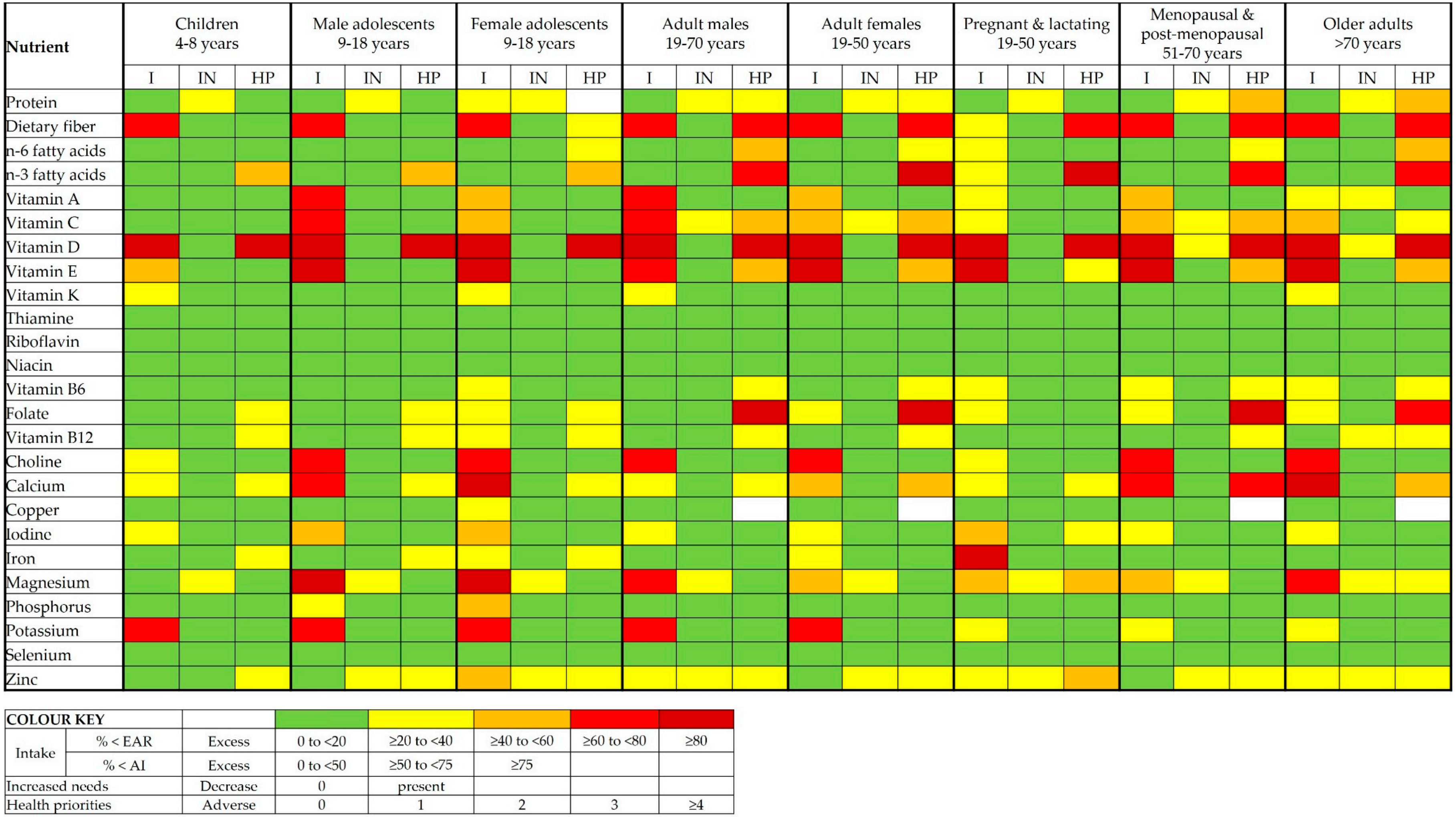

Supplementary Figure S1. Heatmap showing the level of inadequate intake (I), suggested increased need (IN), and number of associations with health priorities (HP) for each included nutrient within each demographic group. Nutrient scoring and therefore the degree of need for prioritization increases from green to dark red.

References

1. USDA Agricultual Research Service. Usual Nutrient Intake from Food and Beverages by Gender and Age, What We Eat in America, NHANES 2017-March 2020 Prepandemic.: United States Department of Agriculture; 2023.
2. USDA Agricultual Research Service. Dietary Fiber (g): Usual Nutrient Intakes from Food and Water, 2003-2006, Compared to Adequate Intakes 2006 [Available from: https://www.ars.usda.gov/ARSUserFiles/80400530/pdf/usual/usual_nutrient_intake_dietary_fiber_2003-06.pdf.
3. USDA Agricultual Research Service. What We Eat in America, NHANES 2005-2006. Usual Nutrient Intakes from Food and Water Compared to 1997 Dietary Reference Intakes for Vitamin D, Calcium, Phosphorus, and Magnesium: US Department of Agriculture; 2009 [Available from: https://www.ars.usda.gov/ARSUserFiles/80400530/pdf/usual/usual_nutrient_intake_vitD_ca_phos_mg_2005-06.pdf.
4. Bailey RL, Pac SG, Fulgoni VL, III, Reidy KC, Catalano PM. Estimation of Total Usual Dietary Intakes of Pregnant Women in the United States. *JAMA Network Open*. **2019**;2(6):e195967-e.
5. Higgins KA, Bi X, Davis BJ, Barraj LM, Scrafford CG, Murphy MM. Adequacy of total usual micronutrient intakes among pregnant women in the United States by level of dairy consumption, NHANES 2003-2016. *Nutr Health*. **2022**:2601060211072325.
6. Pratt N, Durham H, Sherry C. Nutrient Intakes from Food of Lactating Women Do Not Meet Many Dietary Recommendations Important for Infant Development and Maternal Health. *Food and Nutrition Sciences*. **2014**;05:1644-51.
7. Sun H, Weaver CM. Iodine Intake Trends in United States Girls and Women between 2011 and 2020. *The Journal of Nutrition*. **2024**;154(3):928-39.
8. Zhang Z, Fulgoni VL, Kris-Etherton PM, Mitmesser SH. Dietary Intakes of EPA and DHA Omega-3 Fatty Acids among US Childbearing-Age and Pregnant Women: An Analysis of NHANES 2001-2014. *Nutrients*. **2018**;10(4).
9. Institute of Medicine. Dietary Reference Intakes for Calcium and Vitamin D Washington, DC; 2011.
10. National Academies Institute of Medicine. Dietary Reference Intakes: The Essential Guide to Nutrient Requirements. Washington, DC: The National Academies; 2006.
11. National Academies of Science Engineering and Medicine. Dietary Reference Intakes for Sodium and Potassium. Washington, DC; 2019.
12. Hudson JL, Baum JI, Diaz EC, Børsheim E. Dietary Protein Requirements in Children: Methods for Consideration. *Nutrients*. **2021**;13(5).
13. Weiler M, Hertzler SR, Dvoretskiy S. Is It Time to Reconsider the U.S. Recommendations for Dietary Protein and Amino Acid Intake? *Nutrients*. **2023**;15(4).
14. Abrams SA, Chen Z, Hawthorne KM. Magnesium Metabolism in 4-Year-Old to 8-Year-Old Children. *Journal of Bone and Mineral Research*. **2014**;29(1):118-22.
15. Armah SM. Fractional zinc absorption for men, women, and adolescents is overestimated in the current dietary reference intakes. *The Journal of nutrition*. **2016**;146(6):1276-80.
16. Elango R, Humayun MA, Ball RO, Pencharz PB. Evidence that protein requirements have been significantly underestimated. *Curr Opin Clin Nutr Metab Care*. **2010**;13(1):52-7.
17. Humayun MA, Elango R, Ball RO, Pencharz PB. Reevaluation of the protein requirement in young men with the indicator amino acid oxidation technique. *Am J Clin Nutr*. **2007**;86(4):995-1002.
18. Nielsen FH. The Problematic Use of Dietary Reference Intakes to Assess Magnesium Status and Clinical Importance. *Biol Trace Elem Res*. **2019**;188(1):52-9.
19. Hujoel PP, Hujoel MLA. Vitamin C and scar strength: analysis of a historical trial and implications for collagen-related pathologies. *Am J Clin Nutr*. **2022**;115(1):8-17.
20. Levine M, Conry-Cantilena C, Wang Y, Welch RW, Washko PW, Dhariwal KR, et al. Vitamin C pharmacokinetics in healthy volunteers: evidence for a recommended dietary allowance. *Proceedings of the National Academy of Sciences*. **1996**;93(8):3704-9.
21. Montgomery SC, Streit SM, Beebe ML, Maxwell IV PJ. Micronutrient needs of the elderly. *Nutrition in clinical practice*. **2014**;29(4):435-44.
22. Baum JI, Kim I-Y, Wolfe RR. Protein Consumption and the Elderly: What Is the Optimal Level of Intake? *Nutrients*. **2016**;8(6):359.

23. ter Borg S, Verlaan S, Hemsworth J, Mijnarends DM, Schols JM, Luiking YC, de Groot LC. Micronutrient intakes and potential inadequacies of community-dwelling older adults: a systematic review. *British Journal of Nutrition*. **2015**;113(8):1195-206.
24. Chernoff R. Micronutrient requirements in older women. *The American journal of clinical nutrition*. **2005**;81(5):1240S-5S.
25. Cormick G, Betran AP, Romero IB, Cormick MS, Belizán JM, Bardach A, Ciapponi A. Effect of Calcium Fortified Foods on Health Outcomes: A Systematic Review and Meta-Analysis. *Nutrients*. **2021**;13(2).
26. Winzenberg T, Shaw K, Fryer J, Jones G. Effects of calcium supplementation on bone density in healthy children: meta-analysis of randomised controlled trials. *Bmj*. **2006**;333(7572):775.
27. Meli AM, Ali A, Mhd Jalil AM, Mohd Yusof H, Tan MMC. Effects of Physical Activity and Micronutrients on Cognitive Performance in Children Aged 6 to 11 Years: A Systematic Review and Meta-Analysis of Randomized Controlled Trials. *Medicina (Kaunas)*. **2021**;58(1).
28. Tsang BL, Holsted E, McDonald CM, Brown KH, Black R, Mbuya MNN, et al. Effects of Foods Fortified with Zinc, Alone or Cofortified with Multiple Micronutrients, on Health and Functional Outcomes: A Systematic Review and Meta-Analysis. *Adv Nutr*. **2021**;12(5):1821-37.
29. Prades N, Varela E, Flamarique I, Deulofeu R, Baeza I. Water-soluble vitamin insufficiency, deficiency and supplementation in children and adolescents with a psychiatric disorder: a systematic review and meta-analysis. *Nutr Neurosci*. **2023**;26(2):85-107.
30. Winzenberg T, Powell S, Shaw KA, Jones G. Effects of vitamin D supplementation on bone density in healthy children: systematic review and meta-analysis. *Bmj*. **2011**;342:c7254.
31. Winzenberg TM, Powell S, Shaw KA, Jones G. Vitamin D supplementation for improving bone mineral density in children. *Cochrane Database Syst Rev*. **2010**(10):Cd006944.
32. Wu F, Fuleihan GE, Cai G, Lamberg-Allardt C, Viljakainen HT, Rahme M, et al. Vitamin D supplementation for improving bone density in vitamin D-deficient children and adolescents: systematic review and individual participant data meta-analysis of randomized controlled trials. *Am J Clin Nutr*. **2023**;118(3):498-506.
33. Al Khalifah R, Alsheikh R, Alnasser Y, Alsheikh R, Alhelali N, Naji A, Al Backer N. The impact of vitamin D food fortification and health outcomes in children: a systematic review and meta-regression. *Syst Rev*. **2020**;9(1):144.
34. Li B, Xu Y, Zhang X, Zhang L, Wu Y, Wang X, Zhu C. The effect of vitamin D supplementation in treatment of children with autism spectrum disorder: a systematic review and meta-analysis of randomized controlled trials. *Nutr Neurosci*. **2022**;25(4):835-45.
35. Li Q, Zhou Q, Zhang G, Tian X, Li Y, Wang Z, et al. Vitamin D Supplementation and Allergic Diseases during Childhood: A Systematic Review and Meta-Analysis. *Nutrients*. **2022**;14(19).
36. Shah VP, Nayfeh T, Alsawaf Y, Saadi S, Farah M, Zhu Y, et al. A Systematic Review Supporting the Endocrine Society Clinical Practice Guidelines on Vitamin D. *J Clin Endocrinol Metab*. **2024**;109(8):1961-74.
37. Gou H, Wang Y, Liu Y, Peng C, He W, Sun X. Efficacy of vitamin D supplementation on child and adolescent overweight/obesity: a systematic review and meta-analysis of randomized controlled trials. *Eur J Pediatr*. **2023**;182(1):255-64.
38. Soltani S, Beigrezaei S, Abdollahi S, Clark CCT, Ashoori M. Oral vitamin D supplementation and body weight in children and adolescents: a systematic review and meta-analysis of randomized controlled trials. *Eur J Pediatr*. **2023**;182(5):1977-89.
39. Głąbska D, Kołota A, Lachowicz K, Skolmowska D, Stachoń M, Guzek D. Vitamin D Supplementation and Mental Health in Inflammatory Bowel Diseases and Irritable Bowel Syndrome Patients: A Systematic Review. *Nutrients*. **2021**;13(10).
40. Emery S, Häberling I, Berger G, Walitza S, Schmeck K, Albert T, et al. Omega-3 and its domain-specific effects on cognitive test performance in youths: A meta-analysis. *Neurosci Biobehav Rev*. **2020**;112:420-36.
41. van der Wurff ISM, Meyer BJ, de Groot RHM. Effect of Omega-3 Long Chain Polyunsaturated Fatty Acids (n-3 LCPUFA) Supplementation on Cognition in Children and Adolescents: A Systematic Literature Review with a Focus on n-3 LCPUFA Blood Values and Dose of DHA and EPA. *Nutrients*. **2020**;12(10).
42. Lam C, Han L, McIntyre RS, Teopiz KM, Cao B. Comparative Efficacy of Omega-3 Fatty Acid with Other Interventions for Depression in Children and Adolescents: A Systematic Review and Network Meta-Analysis. *J Child Adolesc Psychopharmacol*. **2024**;34(7):282-91.

43. Chen Z, Peng C, Mei J, Zhu L, Kong H. Vitamin D can safely reduce asthma exacerbations among corticosteroid-using children and adults with asthma: a systematic review and meta-analysis of randomized controlled trials. *Nutr Res*. **2021**;92:49-61.
44. Liu M, Wang J, Sun X. A Meta-Analysis on Vitamin D Supplementation and Asthma Treatment. *Front Nutr*. **2022**;9:860628.
45. Guo XF, Zhao T, Han JM, Li S, Li D. Vitamin D and liver cancer risk: A meta-analysis of prospective studies. *Asia Pac J Clin Nutr*. **2020**;29(1):175-82.
46. Song D, Deng Y, Liu K, Zhou L, Li N, Zheng Y, et al. Vitamin D intake, blood vitamin D levels, and the risk of breast cancer: a dose-response meta-analysis of observational studies. *Aging (Albany NY)*. **2019**;11(24):12708-32.
47. Song Y, Lu H, Cheng Y. To identify the association between dietary vitamin D intake and serum levels and risk or prognostic factors for melanoma-systematic review and meta-analysis. *BMJ Open*. **2022**;12(8):e052442.
48. Wu J, Yang N, Yuan M. Dietary and circulating vitamin D and risk of renal cell carcinoma: a meta-analysis of observational studies. *Int Braz J Urol*. **2021**;47(4):733-44.
49. Nguyen NTK, Fan HY, Tsai MC, Tung TH, Huynh QTV, Huang SY, Chen YC. Nutrient Intake through Childhood and Early Menarche Onset in Girls: Systematic Review and Meta-Analysis. *Nutrients*. **2020**;12(9).
50. Guzek D, Kołota A, Lachowicz K, Skolmowska D, Stachoń M, Głąbska D. Influence of Vitamin D Supplementation on Mental Health in Diabetic Patients: A Systematic Review. *Nutrients*. **2021**;13(11).
51. Guzek D, Kołota A, Lachowicz K, Skolmowska D, Stachoń M, Głąbska D. Effect of Vitamin D Supplementation on Depression in Adults: A Systematic Review of Randomized Controlled Trials (RCTs). *Nutrients*. **2023**;15(4).
52. Mikola T, Marx W, Lane MM, Hockey M, Loughman A, Rajapolvi S, et al. The effect of vitamin D supplementation on depressive symptoms in adults: A systematic review and meta-analysis of randomized controlled trials. *Crit Rev Food Sci Nutr*. **2023**;63(33):11784-801.
53. Srifuengfung M, Srifuengfung S, Pummangura C, Pattanaseri K, Oon-Arom A, Srisurapanont M. Efficacy and acceptability of vitamin D supplements for depressed patients: A systematic review and meta-analysis of randomized controlled trials. *Nutrition*. **2023**;108:111968.
54. Wang R, Xu F, Xia X, Xiong A, Dai D, Ling Y, et al. The effect of vitamin D supplementation on primary depression: A meta-analysis. *J Affect Disord*. **2024**;344:653-61.
55. Dominguez LJ, Veronese N, Marrone E, Di Palermo C, Iommi C, Ruggirello R, et al. Vitamin D and Risk of Incident Type 2 Diabetes in Older Adults: An Updated Systematic Review and Meta-Analysis. *Nutrients*. **2024**;16(11).
56. Musazadeh V, Zarezadeh M, Ghalichi F, Kalajahi FH, Ghoreishi Z. Vitamin D supplementation positively affects anthropometric indices: Evidence obtained from an umbrella meta-analysis. *Front Nutr*. **2022**;9:980749.
57. Qi KJ, Zhao ZT, Zhang W, Yang F. The impacts of vitamin D supplementation in adults with metabolic syndrome: A systematic review and meta-analysis of randomized controlled trials. *Front Pharmacol*. **2022**;13:1033026.
58. Arayici ME, Basbinar Y, Ellidokuz H. Vitamin D Intake, Serum 25-Hydroxyvitamin-D (25(OH)D) Levels, and Cancer Risk: A Comprehensive Meta-Meta-Analysis Including Meta-Analyses of Randomized Controlled Trials and Observational Epidemiological Studies. *Nutrients*. **2023**;15(12).
59. Ismail NH, Mussa A, Al-Khreisat MJ, Mohamed Yusoff S, Husin A, Johan MF, Islam MA. The Global Prevalence of Vitamin D Deficiency and Insufficiency in Patients with Multiple Myeloma: A Systematic Review and Meta-Analysis. *Nutrients*. **2023**;15(14).
60. Jung S, Jin S, Je Y. Vitamin D Intake, Blood 25-Hydroxyvitamin D, and Risk of Ovarian Cancer: A Meta-Analysis of Observational Studies. *J Womens Health (Larchmt)*. **2023**;32(5):561-73.
61. Chen QY, Kim S, Lee B, Jeong G, Lee DH, Keum N, et al. Post-Diagnosis Vitamin D Supplement Use and Survival among Cancer Patients: A Meta-Analysis. *Nutrients*. **2022**;14(16).
62. Fu J, Sun J, Zhang C. Vitamin D supplementation and risk of stroke: A meta-analysis of randomized controlled trials. *Front Neurol*. **2022**;13:970111.
63. Khan SU, Khan MU, Riaz H, Valavoor S, Zhao D, Vaughan L, et al. Effects of Nutritional Supplements and Dietary Interventions on Cardiovascular Outcomes: An Umbrella Review and Evidence Map. *Ann Intern Med*. **2019**;171(3):190-8.
64. Pei YY, Zhang Y, Peng XC, Liu ZR, Xu P, Fang F. Association of Vitamin D Supplementation with Cardiovascular Events: A Systematic Review and Meta-Analysis. *Nutrients*. **2022**;14(15).
65. Zhang H, Wang P, Jie Y, Sun Y, Wang X, Fan Y. Predictive value of 25-hydroxyvitamin D level in patients with coronary artery disease: A meta-analysis. *Front Nutr*. **2022**;9:984487.

66. Jayedi A, Daneshvar M, Jibril AT, Sluyter JD, Waterhouse M, Romero BD, et al. Serum 25(OH)D Concentration, Vitamin D Supplementation, and Risk of Cardiovascular Disease and Mortality in Patients with Type 2 Diabetes or Prediabetes: a Systematic Review and Dose-Response Meta-Analysis. *Am J Clin Nutr*. **2023**;118(3):697-707.
67. Mattumpuram J, Maniya MT, Faruqui SK, Ahmed A, Jaiswal V, Harshakumar SP. Cardiovascular and Cerebrovascular Outcomes With Vitamin D Supplementation: A Systematic Review and Meta-Analysis. *Curr Probl Cardiol*. **2024**;49(1 Pt C):102119.
68. Qi S, Luo X, Liu S, Ling B, Si M, Jin H. Effect of vitamin B(2), vitamin C, vitamin D, vitamin E and folic acid in adults with essential hypertension: a systematic review and network meta-analysis. *BMJ Open*. **2024**;14(1):e074511.
69. Rasouli MA, Darvishzadehdaledari S, Alizadeh Z, Moradi G, Gholami F, Mahmoudian A. Vitamin D Supplementation and Cardiovascular Disease Risks in More Than 134000 Individuals in 29 Randomized Clinical Trials and 157000 Individuals in 30 Prospective Cohort Studies: An Updated Systematic Review and Meta-analysis. *J Res Health Sci*. **2023**;23(4):e00594.
70. Serra MO, de Macedo LR, Silva M, Lautner RQ. Effect of Vitamin D supplementation on blood pressure in hypertensive individuals with hypovitaminosis D: a systematic review and meta-analysis. *J Hypertens*. **2024**;42(4):594-604.
71. Xiong J, Zhao C, Li J, Li Y. A systematic review and meta-analysis of the linkage between low vitamin D and the risk as well as the prognosis of stroke. *Brain Behav*. **2024**;14(6):e3577.
72. Arsenyadis F, Ahmad E, Redman E, Yates T, Davies M, Khunti K. The Effects of Omega-3 Supplementation on Depression in Adults with Cardiometabolic Disease: A Systematic Review of Randomised Control Trials. *Nutrients*. **2022**;14(9).
73. Bafkar N, Zeraattalab-Motlagh S, Jayedi A, Shab-Bidar S. Efficacy and safety of omega-3 fatty acids supplementation for anxiety symptoms: a systematic review and dose-response meta-analysis of randomized controlled trials. *BMC Psychiatry*. **2024**;24(1):455.
74. Kelaiditis CF, Gibson EL, Dyall SC. Effects of long-chain omega-3 polyunsaturated fatty acids on reducing anxiety and/or depression in adults; A systematic review and meta-analysis of randomised controlled trials. *Prostaglandins Leukot Essent Fatty Acids*. **2023**;192:102572.
75. Luo XD, Feng JS, Yang Z, Huang QT, Lin JD, Yang B, et al. High-dose omega-3 polyunsaturated fatty acid supplementation might be more superior than low-dose for major depressive disorder in early therapy period: a network meta-analysis. *BMC Psychiatry*. **2020**;20(1):248.
76. Norouziasl R, Zeraattalab-Motlagh S, Jayedi A, Shab-Bidar S. Efficacy and safety of n-3 fatty acids supplementation on depression: a systematic review and dose-response meta-analysis of randomised controlled trials. *Br J Nutr*. **2024**;131(4):658-71.
77. Jiang H, Wang L, Wang D, Yan N, Li C, Wu M, et al. Omega-3 polyunsaturated fatty acid biomarkers and risk of type 2 diabetes, cardiovascular disease, cancer, and mortality. *Clin Nutr*. **2022**;41(8):1798-807.
78. Lu Y, Li D, Wang L, Zhang H, Jiang F, Zhang R, et al. Comprehensive Investigation on Associations between Dietary Intake and Blood Levels of Fatty Acids and Colorectal Cancer Risk. *Nutrients*. **2023**;15(3).
79. Wang Y, Liu K, Long T, Long J, Li Y, Li J, Cheng L. Dietary fish and omega-3 polyunsaturated fatty acids intake and cancer survival: A systematic review and meta-analysis. *Crit Rev Food Sci Nutr*. **2023**;63(23):6235-51.
80. Abdelhamid AS, Brown TJ, Brainard JS, Biswas P, Thorpe GC, Moore HJ, et al. Omega-3 fatty acids for the primary and secondary prevention of cardiovascular disease. *Cochrane Database Syst Rev*. **2020**;3(3):Cd003177.
81. Bernasconi AA, Wiest MM, Lavie CJ, Milani RV, Laukkanen JA. Effect of Omega-3 Dosage on Cardiovascular Outcomes: An Updated Meta-Analysis and Meta-Regression of Interventional Trials. *Mayo Clin Proc*. **2021**;96(2):304-13.
82. Marston NA, Giugliano RP, Im K, Silverman MG, O'Donoghue ML, Wiviott SD, et al. Association Between Triglyceride Lowering and Reduction of Cardiovascular Risk Across Multiple Lipid-Lowering Therapeutic Classes: A Systematic Review and Meta-Regression Analysis of Randomized Controlled Trials. *Circulation*. **2019**;140(16):1308-17.
83. Sohouli MH, Roshan MM, Olusola OF, Fatahi S, Omidi HR, Sharifi P, et al. Impact of Omega-3 supplementation on homocysteine levels in humans: A systematic review and meta-regression analysis of randomized controlled trials. *Nutr Metab Cardiovasc Dis*. **2022**;32(9):2013-25.
84. Xu Q, Du L, Gu H, Ji M, Zhan L. The effect of omega-3 polyunsaturated fatty acids on stroke treatment and prevention: a systematic review and meta-analysis. *Nutr Hosp*. **2022**;39(4):924-35.

85. Yokoyama Y, Kuno T, Morita SX, Slipczuk L, Takagi H, Briasoulis A, et al. Eicosapentaenoic Acid for Cardiovascular Events Reduction- Systematic Review and Network Meta-Analysis of Randomized Controlled Trials. *J Cardiol*. **2022**;80(5):416-22.
86. Yu F, Qi S, Ji Y, Wang X, Fang S, Cao R. Effects of omega-3 fatty acid on major cardiovascular outcomes: A systematic review and meta-analysis. *Medicine (Baltimore)*. **2022**;101(30):e29556.
87. Luo S, Hou H, Wang Y, Li Y, Zhang L, Zhang H, et al. Effects of omega-3, omega-6, and total dietary polyunsaturated fatty acid supplementation in patients with atherosclerotic cardiovascular disease: a systematic review and meta-analysis. *Food Funct*. **2024**;15(3):1208-22.
88. Wang T, Zhang X, Zhou N, Shen Y, Li B, Chen BE, Li X. Association Between Omega-3 Fatty Acid Intake and Dyslipidemia: A Continuous Dose-Response Meta-Analysis of Randomized Controlled Trials. *J Am Heart Assoc*. **2023**;12(11):e029512.
89. Yan J, Liu M, Yang D, Zhang Y, An F. Efficacy and Safety of Omega-3 Fatty Acids in the Prevention of Cardiovascular Disease: A Systematic Review and Meta-analysis. *Cardiovasc Drugs Ther*. **2024**;38(4):799-817.
90. Yin S, Xu H, Xia J, Lu Y, Xu D, Sun J, et al. Effect of Alpha-Linolenic Acid Supplementation on Cardiovascular Disease Risk Profile in Individuals with Obesity or Overweight: A Systematic Review and Meta-Analysis of Randomized Controlled Trials. *Adv Nutr*. **2023**;14(6):1644-55.
91. Arabi SM, Bahari H, Chambari M, Bahrami LS, Mohaildeen Gubari MI, Watts GF, Sahebkar A. Omega-3 fatty acids and endothelial function: A GRADE-assessed systematic review and meta-analysis. *Eur J Clin Invest*. **2024**;54(2):e14109.
92. Lee YS, Park JW, Joo M, Moon S, Kim K, Kim MG. Effects of Omega-3 Fatty Acids on Flow-mediated Dilatation and Carotid Intima Media Thickness: A Meta-analysis. *Curr Atheroscler Rep*. **2023**;25(10):629-41.
93. Yosaee S, Keshtkaran Z, Abdollahi S, Shidfar F, Sarris J, Soltani S. The effect of vitamin C supplementation on mood status in adults: a systematic review and meta-analysis of randomized controlled clinical trials. *Gen Hosp Psychiatry*. **2021**;71:36-42.
94. Ashor AW, Siervo M, Lara J, Oggioni C, Afshar S, Mathers JC. Effect of vitamin C and vitamin E supplementation on endothelial function: a systematic review and meta-analysis of randomised controlled trials. *British Journal of Nutrition*. **2015**;113(8):1182-94.
95. Jayedi A, Rashidy-Pour A, Parohan M, Zargar MS, Shab-Bidar S. Dietary and circulating vitamin C, vitamin E, β-carotene and risk of total cardiovascular mortality: a systematic review and dose-response meta-analysis of prospective observational studies. *Public Health Nutr*. **2019**;22(10):1872-87.
96. Emami MR, Safabakhsh M, Alizadeh S, Asbaghi O, Khosroshahi MZ. Effect of vitamin E supplementation on blood pressure: a systematic review and meta-analysis. *Journal of Human Hypertension*. **2019**;33(7):499-507.
97. Xiong Z, Liu L, Jian Z, Ma Y, Li H, Jin X, et al. Vitamin E and Multiple Health Outcomes: An Umbrella Review of Meta-Analyses. *Nutrients*. **2023**;15(15).
98. Altaf R, Gonzalez I, Rubino K, Nemec EC, 2nd. Folate as adjunct therapy to SSRI/SNRI for major depressive disorder: Systematic review & meta-analysis. *Complement Ther Med*. **2021**;61:102770.
99. Lam NSK, Long XX, Li X, Saad M, Lim F, Doery JC, et al. The potential use of folate and its derivatives in treating psychiatric disorders: A systematic review. *Biomed Pharmacother*. **2022**;146:112541.
100. Aydoğdu GS, Akyakar B, Kalaycı Z, Uçar A, Gezmen-Karadağ M. Folic Acid as a Potential Vitamin in Glycemic Control: A Systematic Review. *Curr Nutr Rep*. **2024**.
101. Lei J, Ren F, Li W, Guo X, Liu Q, Gao H, et al. Use of folic acid supplementation to halt and even reverse the progression of gastric precancerous conditions: a meta-analysis. *BMC Gastroenterol*. **2022**;22(1):370.
102. Moazzen S, Dolatkhah R, Tabrizi JS, Shaarbafi J, Alizadeh BZ, de Bock GH, Dastgiri S. Folic acid intake and folate status and colorectal cancer risk: A systematic review and meta-analysis. *Clin Nutr*. **2018**;37(6 Pt A):1926-34.
103. Miao Y, Guo Y, Chen Y, Lin Y, Lu Y, Guo Q. The effect of B-vitamins on the prevention and treatment of cardiovascular diseases: a systematic review and meta-analysis. *Nutr Rev*. **2024**;82(10):1386-401.
104. da Silva LEM, de Santana MLP, Costa PRF, Pereira EM, Nepomuceno CMM, Queiroz VAO, et al. Zinc supplementation combined with antidepressant drugs for treatment of patients with depression: a systematic review and meta-analysis. *Nutr Rev*. **2021**;79(1):1-12.
105. Yosaee S, Clark CCT, Keshtkaran Z, Ashourpour M, Keshani P, Soltani S. Zinc in depression: From development to treatment: A comparative/ dose response meta-analysis of observational studies and randomized controlled trials. *General Hospital Psychiatry*. **2022**;74:110-7.

106. Fu L, Zhang G, Qian S, Zhang Q, Tan M. Associations between dietary fiber intake and cardiovascular risk factors: An umbrella review of meta-analyses of randomized controlled trials. *Front Nutr*. **2022**;9:972399.
107. McRae MP. Dietary Fiber Intake and Type 2 Diabetes Mellitus: An Umbrella Review of Meta-analyses. *Journal of Chiropractic Medicine*. **2018**;17(1):44-53.
108. Reynolds AN, Akerman AP, Mann J. Dietary fibre and whole grains in diabetes management: Systematic review and meta-analyses. *PLoS Med*. **2020**;17(3):e1003053.
109. Ma Y, Hu M, Zhou L, Ling S, Li Y, Kong B, Huang P. Dietary fiber intake and risks of proximal and distal colon cancers: A meta-analysis. *Medicine (Baltimore)*. **2018**;97(36):e11678.
110. Mirrafiei A, Jayedi A, Shab-Bidar S. Total and different dietary fiber subtypes and the risk of all-cause, cardiovascular, and cancer mortality: a dose-response meta-analysis of prospective cohort studies. *Food Funct*. **2023**;14(24):10667-80.
111. Ramezani F, Pourghazi F, Eslami M, Gholami M, Mohammadian Khonsari N, Ejtahed HS, et al. Dietary fiber intake and all-cause and cause-specific mortality: An updated systematic review and meta-analysis of prospective cohort studies. *Clin Nutr*. **2024**;43(1):65-83.
112. Veronese N, Solmi M, Caruso MG, Giannelli G, Osella AR, Evangelou E, et al. Dietary fiber and health outcomes: an umbrella review of systematic reviews and meta-analyses. *Am J Clin Nutr*. **2018**;107(3):436-44.
113. Watling CZ, Wojt A, Florio AA, Butera G, Albanes D, Weinstein SJ, et al. Fiber and whole grain intakes in relation to liver cancer risk: An analysis in 2 prospective cohorts and systematic review and meta-analysis of prospective studies. *Hepatology*. **2024**;80(3):552-65.
114. Kim Y, Je Y. Dietary fibre intake and mortality from cardiovascular disease and all cancers: A meta-analysis of prospective cohort studies. *Arch Cardiovasc Dis*. **2016**;109(1):39-54.
115. Li DB, Hao QQ, Hu HL. The relationship between dietary fibre and stroke: A meta-analysis. *J Stroke Cerebrovasc Dis*. **2023**;32(8):107144.
116. Cormick G, Ciapponi A, Harbron J, Perez SM, Vazquez P, Rivo J, et al. Calcium supplementation for people with overweight or obesity. *Cochrane Database Syst Rev*. **2024**;5(5):Cd012268.
117. Yousefi M, Eshaghian N, Heidarzadeh-Esfahani N, Askari G, Rasekhi H, Sadeghi O. Dietary intake and biomarkers of linoleic acid and risk of prostate cancer in men: A systematic review and dose-response meta-analysis of prospective cohort studies. *Crit Rev Food Sci Nutr*. **2024**;64(24):8553-69.
118. Marklund M, Wu JHY, Imamura F, Del Gobbo LC, Fretts A, de Goede J, et al. Biomarkers of Dietary Omega-6 Fatty Acids and Incident Cardiovascular Disease and Mortality. *Circulation*. **2019**;139(21):2422-36.
119. Ren XL, Liu Y, Chu WJ, Li ZW, Zhang SS, Zhou ZL, et al. Blood levels of omega-6 fatty acids and coronary heart disease: a systematic review and metaanalysis of observational epidemiology. *Crit Rev Food Sci Nutr*. **2023**;63(26):7983-95.
120. Ma Y, Zheng Z, Zhuang L, Wang H, Li A, Chen L, Liu L. Dietary Macronutrient Intake and Cardiovascular Disease Risk and Mortality: A Systematic Review and Dose-Response Meta-Analysis of Prospective Cohort Studies. *Nutrients*. **2024**;16(1).
121. Sohouli MH, Almuqayyid F, Alfardous Alazm A, Ziamanesh F, Izze da Silva Magalhães E, Bagheri SE, et al. A comprehensive review and meta-regression analysis of randomized controlled trials examining the impact of vitamin B12 supplementation on homocysteine levels. *Nutr Rev*. **2024**;82(6):726-37.
122. Zhao H, Mei K, Hu Q, Wu Y, Xu Y, Qinling, et al. Circulating copper levels and the risk of cardio-cerebrovascular diseases and cardiovascular and all-cause mortality: A systematic review and meta-analysis of longitudinal studies. *Environ Pollut*. **2024**;340(Pt 2):122711.
123. Iliuta F, Pijoan JI, Lainz L, Exposito A, Matorras R. Women's vitamin D levels and IVF results: a systematic review of the literature and meta-analysis, considering three categories of vitamin status (replete, insufficient and deficient). *Hum Fertil (Camb)*. **2022**;25(2):228-46.
124. Meng X, Zhang J, Wan Q, Huang J, Han T, Qu T, Yu LL. Influence of Vitamin D supplementation on reproductive outcomes of infertile patients: a systematic review and meta-analysis. *Reprod Biol Endocrinol*. **2023**;21(1):17.
125. Moridi I, Chen A, Tal O, Tal R. The Association between Vitamin D and Anti-Müllerian Hormone: A Systematic Review and Meta-Analysis. *Nutrients*. **2020**;12(6).
126. Abodi M, De Cosmi V, Parazzini F, Agostoni C. Omega-3 fatty acids dietary intake for oocyte quality in women undergoing assisted reproductive techniques: A systematic review. *Eur J Obstet Gynecol Reprod Biol*. **2022**;275:97-105.

127. Ren X, Xu P, Zhang D, Liu K, Song D, Zheng Y, et al. Association of folate intake and plasma folate level with the risk of breast cancer: a dose-response meta-analysis of observational studies. *Aging (Albany NY)*. **2020**;12(21):21355-75.
128. Farvid MS, Spence ND, Holmes MD, Barnett JB. Fiber consumption and breast cancer incidence: A systematic review and meta-analysis of prospective studies. *Cancer*. **2020**;126(13):3061-75.
129. Ghoreishy SM, Bagheri A, Nejad MM, Larijani B, Esmaillzadeh A. Association between calcium intake and risk of breast cancer: An updated systematic review and dose-response meta-analysis of cohort studies. *Clin Nutr ESPEN*. **2023**;55:251-9.
130. Ribamar A, Almeida B, Soares A, Peniche B, Jesus P, Cruz SPD, Ramalho A. Relationship between vitamin D deficiency and both gestational and postpartum depression. *Nutr Hosp*. **2020**;37(6):1238-45.
131. Tan Q, Liu S, Chen D. Poor vitamin D status and the risk of maternal depression: a dose-response meta-analysis of observational studies. *Public Health Nutr*. **2021**;24(8):2161-70.
132. Chan KY, Wong MMH, Pang SSH, Lo KKH. Dietary supplementation for gestational diabetes prevention and management: a meta-analysis of randomized controlled trials. *Arch Gynecol Obstet*. **2021**;303(6):1381-91.
133. Chien MC, Huang CY, Wang JH, Shih CL, Wu P. Effects of vitamin D in pregnancy on maternal and offspring health-related outcomes: An umbrella review of systematic review and meta-analyses. *Nutr Diabetes*. **2024**;14(1):35.
134. Gallo S, McDermid JM, Al-Nimr RI, Hakeem R, Moreschi JM, Pari-Keener M, et al. Vitamin D Supplementation during Pregnancy: An Evidence Analysis Center Systematic Review and Meta-Analysis. *J Acad Nutr Diet*. **2020**;120(5):898-924.e4.
135. Irwinda R, Hiksas R, Lokeswara AW, Wibowo N. Vitamin D supplementation higher than 2000 IU/day compared to lower dose on maternal-fetal outcome: Systematic review and meta-analysis. *Womens Health (Lond)*. **2022**;18:17455057221111066.
136. Palacios C, Kostiuk LL, Cuthbert A, Weeks J. Vitamin D supplementation for women during pregnancy. *Cochrane Database Syst Rev*. **2024**;7(7):Cd008873.
137. Wang M, Chen Z, Hu Y, Wang Y, Wu Y, Lian F, et al. The effects of vitamin D supplementation on glycemic control and maternal-neonatal outcomes in women with established gestational diabetes mellitus: A systematic review and meta-analysis. *Clinical Nutrition*. **2021**;40(5):3148-57.
138. Fogacci S, Fogacci F, Banach M, Michos ED, Hernandez AV, Lip GYH, et al. Vitamin D supplementation and incident preeclampsia: A systematic review and meta-analysis of randomized clinical trials. *Clin Nutr*. **2020**;39(6):1742-52.
139. Gunabalasingam S, De Almeida Lima Slizys D, Quotah O, Magee L, White SL, Rigutto-Farebrother J, et al. Micronutrient supplementation interventions in preconception and pregnant women at increased risk of developing pre-eclampsia: a systematic review and meta-analysis. *Eur J Clin Nutr*. **2023**;77(7):710-30.
140. Wu C, Song Y, Wang X. Vitamin D Supplementation for the Outcomes of Patients with Gestational Diabetes Mellitus and Neonates: A Meta-Analysis and Systematic Review. *Int J Clin Pract*. **2023**;2023:1907222.
141. Bahardoust M, Salari S, Ghotbi N, Rahimpour E, Haghmoradi M, Alipour H, Soleimani M. Association between prenatal vitamin D deficiency with dental caries in infants and children: a systematic review and meta-analysis. *BMC Pregnancy Childbirth*. **2024**;24(1):256.
142. Luo C, Sun Y, Zeng Z, Liu Y, Peng S. Vitamin D supplementation in pregnant women or infants for preventing allergic diseases: a systematic review and meta-analysis of randomized controlled trials. *Chin Med J (Engl)*. **2022a**;135(3):276-84.
143. Shi D, Wang D, Meng Y, Chen J, Mu G, Chen W. Maternal vitamin D intake during pregnancy and risk of asthma and wheeze in children: a systematic review and meta-analysis of observational studies. *J Matern Fetal Neonatal Med*. **2021**;34(4):653-9.
144. Sobczak M, Pawliczak R. Relationship between vitamin D and asthma from gestational to adulthood period: a meta-analysis of randomized clinical trials. *BMC Pulm Med*. **2023**;23(1):212.
145. Bi WG, Nuyt AM, Weiler H, Leduc L, Santamaria C, Wei SQ. Association Between Vitamin D Supplementation During Pregnancy and Offspring Growth, Morbidity, and Mortality: A Systematic Review and Meta-analysis. *JAMA Pediatr*. **2018**;172(7):635-45.
146. Liu Y, Ding C, Xu R, Wang K, Zhang D, Pang W, et al. Effects of vitamin D supplementation during pregnancy on offspring health at birth: A meta-analysis of randomized controlled trails. *Clin Nutr*. **2022**;41(7):1532-40.

147. Luo T, Lin Y, Lu J, Lian X, Guo Y, Han L, Guo Y. Effects of vitamin D supplementation during pregnancy on bone health and offspring growth: A systematic review and meta-analysis of randomized controlled trials. *PLoS One*. **2022b**;17(10):e0276016.
148. Moon RJ, Green HD, D'Angelo S, Godfrey KM, Davies JH, Curtis EM, et al. The effect of pregnancy vitamin D supplementation on offspring bone mineral density in childhood: a systematic review and meta-analysis. *Osteoporos Int*. **2023**;34(7):1269-79.
149. Wu H, Zhang C, Wang Y, Li Y. Does vitamin E prevent asthma or wheeze in children: A systematic review and meta-analysis. *Paediatric Respiratory Reviews*. **2018**;27:60-8.
150. Mocking RJT, Steijn K, Roos C, Assies J, Bergink V, Ruhé HG, Schene AH. Omega-3 Fatty Acid Supplementation for Perinatal Depression: A Meta-Analysis. *J Clin Psychiatry*. **2020**;81(5).
151. Sun J, Wang J, Ma W, Miao M, Sun G. Effects of Additional Dietary Fiber Supplements on Pregnant Women with Gestational Diabetes: A Systematic Review and Meta-Analysis of Randomized Controlled Studies. *Nutrients*. **2022**;14(21).
152. Bakouei F, Delavar MA, Mashayekh-Amiri S, Esmailzadeh S, Taheri Z. Efficacy of n-3 fatty acids supplementation on the prevention of pregnancy induced-hypertension or preeclampsia: A systematic review and meta-analysis. *Taiwan J Obstet Gynecol*. **2020**;59(1):8-15.
153. Liu W, Gao M, Yang S, Sun C, Bi Y, Li Y, et al. Effects of omega-3 supplementation on glucose and lipid metabolism in patients with gestational diabetes: A meta-analysis of randomized controlled trials. *J Diabetes Complications*. **2023**;37(4):108451.
154. Abdelrahman MA, Osama H, Saeed H, Madney YM, Harb HS, Abdelrahim MEA. Impact of n-3 polyunsaturated fatty acid intake in pregnancy on maternal health and birth outcomes: systematic review and meta-analysis from randomized controlled trails. *Arch Gynecol Obstet*. **2023**;307(1):249-62.
155. Bilgundi K, Viswanatha GL, Purushottam KM, John J, Kamath AP, Kishore A, et al. Docosahexaenoic Acid and Pregnancy: A Systematic Review and Meta-Analysis of the Association with Improved Maternal and Fetal Health. *Nutr Res*. **2024**;128:82-93.
156. Li N, Jiang J, Guo L. Effects of maternal folate and vitamin B12 on gestational diabetes mellitus: a dose-response meta-analysis of observational studies. *Eur J Clin Nutr*. **2022**;76(11):1502-12.
157. Chen Z, Xing Y, Yu X, Dou Y, Ma D. Effect of Folic Acid Intake on Infant and Child Allergic Diseases: Systematic Review and Meta-Analysis. *Front Pediatr*. **2020**;8:615406.
158. Gao Y, Sheng C, Xie RH, Sun W, Asztalos E, Moddemann D, et al. New Perspective on Impact of Folic Acid Supplementation during Pregnancy on Neurodevelopment/Autism in the Offspring Children - A Systematic Review. *PLoS One*. **2016**;11(11):e0165626.
159. Partap U, Chowdhury R, Taneja S, Bhandari N, De Costa A, Bahl R, Fawzi W. Preconception and periconception interventions to prevent low birth weight, small for gestational age and preterm birth: a systematic review and meta-analysis. *BMJ Glob Health*. **2022**;7(8).
160. Tan X, Huang Y. Magnesium supplementation for glycemic status in women with gestational diabetes: a systematic review and meta-analysis. *Gynecol Endocrinol*. **2022**;38(3):202-6.
161. Li X, Zhao J. The influence of zinc supplementation on metabolic status in gestational diabetes: a meta-analysis of randomized controlled studies. *J Matern Fetal Neonatal Med*. **2021**;34(13):2140-5.
162. Ceylan MN, Akdas S, Yazihan N. The Effects of Zinc Supplementation on C-Reactive Protein and Inflammatory Cytokines: A Meta-Analysis and Systematical Review. *J Interferon Cytokine Res*. **2021**;41(3):81-101.
163. Hofmeyr GJ, Lawrie TA, Atallah Á N, Torloni MR. Calcium supplementation during pregnancy for preventing hypertensive disorders and related problems. *Cochrane Database Syst Rev*. **2018**;10(10):Cd001059.
164. Jaiswal V, Joshi A, Jha M, Hanif M, Arora A, Gupta S, et al. Association between calcium supplementation and gestational hypertension, and preeclampsia: A Meta-analysis of 26 randomized controlled trials. *Curr Probl Cardiol*. **2024**;49(3):102217.
165. Sun X, Li H, He X, Li M, Yan P, Xun Y, et al. The association between calcium supplement and preeclampsia and gestational hypertension: a systematic review and meta-analysis of randomized trials. *Hypertens Pregnancy*. **2019**;38(2):129-39.
166. Woo Kinshella ML, Sarr C, Sandhu A, Bone JN, Vidler M, Moore SE, et al. Calcium for pre-eclampsia prevention: A systematic review and network meta-analysis to guide personalised antenatal care. *Bjog*. **2022**;129(11):1833-43.
167. Greenwood DC, Webster J, Keeble C, Taylor E, Hardie LJ. Maternal Iodine Status and Birth Outcomes: A Systematic Literature Review and Meta-Analysis. *Nutrients*. **2023**;15(2).

168. Machamba AAL, Azevedo FM, Fracalossi KO, do CCFS. Effect of iodine supplementation in pregnancy on neurocognitive development on offspring in iodine deficiency areas: a systematic review. *Arch Endocrinol Metab*. **2021**;65(3):352-67.
169. Gaugris S, Heaney RP, Boonen S, Kurth H, Bentkover JD, Sen SS. Vitamin D inadequacy among post-menopausal women: a systematic review. *Qjm*. **2005**;98(9):667-76.
170. Habibi Ghahfarrokhi S, Mohammadian-Hafshejani A, Sherwin CMT, Heidari-Soureshjani S. Relationship between serum vitamin D and hip fracture in the elderly: a systematic review and meta-analysis. *J Bone Miner Metab*. **2022**;40(4):541-53.
171. Liu C, Kuang X, Li K, Guo X, Deng Q, Li D. Effects of combined calcium and vitamin D supplementation on osteoporosis in postmenopausal women: a systematic review and meta-analysis of randomized controlled trials. *Food Funct*. **2020**;11(12):10817-27.
172. Reis AR, Santos RKF, Dos Santos CB, Santos BDC, de Carvalho GB, Brandão-Lima PN, et al. Supplementation of vitamin D isolated or calcium-associated with bone remodeling and fracture risk in postmenopausal women without osteoporosis: A systematic review of randomized clinical trials. *Nutrition*. **2023**;116:112151.
173. Decandia D, Landolfo E, Sacchetti S, Gelfo F, Petrosini L, Cutuli D. n-3 PUFA Improve Emotion and Cognition during Menopause: A Systematic Review. *Nutrients*. **2022**;14(9).
174. Iqbal AZ, Wu SK, Zailani H, Chiu WC, Liu WC, Su KP, Lee SD. Effects of Omega-3 Polyunsaturated Fatty Acids Intake on Vasomotor Symptoms, Sleep Quality and Depression in Postmenopausal Women: A Systematic Review. *Nutrients*. **2023**;15(19).
175. Bristow SM, Bolland MJ, Gamble GD, Leung W, Reid IR. Dietary calcium intake and change in bone mineral density in older adults: a systematic review of longitudinal cohort studies. *Eur J Clin Nutr*. **2022**;76(2):196-205.
176. Wu J, Xu L, Lv Y, Dong L, Zheng Q, Li L. Quantitative analysis of efficacy and associated factors of calcium intake on bone mineral density in postmenopausal women. *Osteoporos Int*. **2017**;28(6):2003-10.
177. Shams-White MM, Chung M, Du M, Fu Z, Insogna KL, Karlsen MC, et al. Dietary protein and bone health: a systematic review and meta-analysis from the National Osteoporosis Foundation. *The American Journal of Clinical Nutrition*. **2017**;105(6):1528-43.
178. Chang J, Liu M, Liu C, Zhou S, Jiao Y, Sun H, Ji Y. Effects of vitamins and polyunsaturated fatty acids on cognitive function in older adults with mild cognitive impairment: a meta-analysis of randomized controlled trials. *Eur J Nutr*. **2024**;63(4):1003-22.
179. He X, Yu H, Fang J, Qi Z, Pei S, Yan B, et al. The effect of n-3 polyunsaturated fatty acid supplementation on cognitive function outcomes in the elderly depends on the baseline omega-3 index. *Food Funct*. **2023**;14(21):9506-17.
180. Stanfar K, Hawes C, Ghajar M, Byham-Gray L, Radler DR. Diet modification reduces pain and improves function in adults with osteoarthritis: a systematic review. *J Hum Nutr Diet*. **2024**;37(4):847-84.
181. Suh SW, Lim E, Burm SY, Lee H, Bae JB, Han JW, Kim KW. The influence of n-3 polyunsaturated fatty acids on cognitive function in individuals without dementia: a systematic review and dose-response meta-analysis. *BMC Med*. **2024**;22(1):109.
182. Timraz M, Binmahfoz A, Quinn TJ, Combet E, Gray SR. The Effect of Long Chain n-3 Fatty Acid Supplementation on Muscle Strength in Older Adults: A Systematic Review and Meta-Analysis. *Nutrients*. **2023**;15(16).
183. Tseng PT, Zeng BY, Zeng BS, Liao YC, Stubbs B, Kuo JS, et al. Omega-3 polyunsaturated fatty acids in sarcopenia management: A network meta-analysis of randomized controlled trials. *Ageing Res Rev*. **2023**;90:102014.
184. Uchida Y, Tsuji K, Ochi E. Effects of Omega-3 fatty acids supplementation and resistance training on skeletal muscle. *Clin Nutr ESPEN*. **2024**;61:189-96.
185. Yang L, Zhao F, Sun Y, Wang Z, Li Q, Wang H, Lu Y. N-3 Polyunsaturated Fatty Acids in Elderly with Mild Cognitive Impairment: A Systemic Review and Meta-Analysis. *J Alzheimers Dis*. **2024**;99(s1):S81-s95.
186. Cornish SM, Cordingley DM, Shaw KA, Forbes SC, Leonhardt T, Bristol A, et al. Effects of Omega-3 Supplementation Alone and Combined with Resistance Exercise on Skeletal Muscle in Older Adults: A Systematic Review and Meta-Analysis. *Nutrients*. **2022**;14(11).
187. Rondanelli M, Perna S, Riva A, Petrangolini G, Di Paolo E, Gasparri C. Effects of n-3 EPA and DHA supplementation on fat free mass and physical performance in elderly. A systematic review and meta-analysis of randomized clinical trial. *Mech Ageing Dev*. **2021**;196:111476.

188. Wei BZ, Li L, Dong CW, Tan CC, Xu W. The Relationship of Omega-3 Fatty Acids with Dementia and Cognitive Decline: Evidence from Prospective Cohort Studies of Supplementation, Dietary Intake, and Blood Markers. *Am J Clin Nutr*. **2023**;117(6):1096-109.
189. D'Ecclesiis O, Gavioli C, Martinoli C, Raimondi S, Chiocca S, Miccolo C, et al. Vitamin D and SARS-CoV2 infection, severity and mortality: A systematic review and meta-analysis. *PLoS One*. **2022**;17(7):e0268396.
190. Chen WY, Cheng YC, Chiu CC, Liu HC, Huang MC, Tu YK, Kuo PH. Effects of Vitamin D Supplementation on Cognitive Outcomes: A Systematic Review and Meta-Analysis. *Neuropsychol Rev*. **2024**;34(2):568-80.
191. Xiong A, Li H, Lin M, Xu F, Xia X, Dai D, et al. Effects of active vitamin D analogues on muscle strength and falls in elderly people: an updated meta-analysis. *Front Endocrinol (Lausanne)*. **2024**;15:1327623.
192. Bergink AP, Zillikens MC, Van Leeuwen JP, Hofman A, Uitterlinden AG, van Meurs JB. 25-Hydroxyvitamin D and osteoarthritis: A meta-analysis including new data. *Semin Arthritis Rheum*. **2016**;45(5):539-46.
193. Cao Y, Winzenberg T, Nguo K, Lin J, Jones G, Ding C. Association between serum levels of 25-hydroxyvitamin D and osteoarthritis: a systematic review. *Rheumatology (Oxford)*. **2013**;52(7):1323-34.
194. Hung KC, Wang LK, Lin YT, Yu CH, Chang CY, Sun CK, Chen JY. Association of preoperative vitamin D deficiency with the risk of postoperative delirium and cognitive dysfunction: A meta-analysis. *J Clin Anesth*. **2022**;79:110681.
195. Tan L, He R, Zheng X. Effect of vitamin D, calcium, or combined supplementation on fall prevention: a systematic review and updated network meta-analysis. *BMC Geriatr*. **2024**;24(1):390.
196. Wang K, Xia C, Zhou L, Zheng Y, Wang X, Cheng L. The Association between Vitamin D Deficiency and the Risk of Mortality after Hip Fractures: A Systematic Review and Meta-Analysis. *J Nutr Sci Vitaminol (Tokyo)*. **2024**;70(2):89-97.
197. de Souza MM, Moraes Dantas RL, Leão Durães V, Defante MLR, Mendes TB. Vitamin D Supplementation and the Incidence of Fractures in the Elderly Healthy Population: A Meta-analysis of Randomized Controlled Trials. *J Gen Intern Med*. **2024**.
198. Prokopidis K, Giannos P, Katsikas Triantafyllidis K, Kechagias KS, Mesinovic J, Witard OC, Scott D. Effect of vitamin D monotherapy on indices of sarcopenia in community-dwelling older adults: a systematic review and meta-analysis. *J Cachexia Sarcopenia Muscle*. **2022**;13(3):1642-52.
199. Huang L, Zhao J, Chen Y, Ma F, Huang G, Li W. Baseline folic acid status affects the effectiveness of folic acid supplements in cognitively relevant outcomes in older adults: a systematic review. *Aging Ment Health*. **2022**;26(3):457-63.
200. Li S, Guo Y, Men J, Fu H, Xu T. The preventive efficacy of vitamin B supplements on the cognitive decline of elderly adults: a systematic review and meta-analysis. *BMC Geriatr*. **2021**;21(1):367.
201. Wang Z, Zhu W, Xing Y, Jia J, Tang Y. B vitamins and prevention of cognitive decline and incident dementia: a systematic review and meta-analysis. *Nutr Rev*. **2022**;80(4):931-49.
202. Zhang X, Bao G, Liu D, Yang Y, Li X, Cai G, et al. The Association Between Folate and Alzheimer's Disease: A Systematic Review and Meta-Analysis. *Front Neurosci*. **2021**;15:661198.
203. Nasimi N, Sohrabi Z, Nunes EA, Sadeghi E, Jamshidi S, Gholami Z, et al. Whey Protein Supplementation with or without Vitamin D on Sarcopenia-Related Measures: A Systematic Review and Meta-Analysis. *Adv Nutr*. **2023**;14(4):762-73.
204. Bai GH, Tsai MC, Tsai HW, Chang CC, Hou WH. Effects of branched-chain amino acid-rich supplementation on EWGSOP2 criteria for sarcopenia in older adults: a systematic review and meta-analysis. *Eur J Nutr*. **2022**;61(2):637-51.
205. Camargo LDR, Doneda D, Oliveira VR. Whey protein ingestion in elderly diet and the association with physical, performance and clinical outcomes. *Exp Gerontol*. **2020**;137:110936.
206. Hengeveld LM, de Goede J, Afman LA, Bakker SJL, Beulens JWJ, Blaak EE, et al. Health Effects of Increasing Protein Intake Above the Current Population Reference Intake in Older Adults: A Systematic Review of the Health Council of the Netherlands. *Adv Nutr*. **2022**;13(4):1083-117.
207. Morgan PT, Harris DO, Marshall RN, Quinlan JI, Edwards SJ, Allen SL, Breen L. Protein Source and Quality for Skeletal Muscle Anabolism in Young and Older Adults: A Systematic Review and Meta-Analysis. *J Nutr*. **2021**;151(7):1901-20.
208. Negm AM, Lee J, Hamidian R, Jones CA, Khadaroo RG. Management of Sarcopenia: A Network Meta-Analysis of Randomized Controlled Trials. *J Am Med Dir Assoc*. **2022**;23(5):707-14.

209. Rus GE, Porter J, Brunton A, Crocker M, Kotsimbos Z, Percic J, et al. Nutrition interventions implemented in hospital to lower risk of sarcopenia in older adults: A systematic review of randomised controlled trials. *Nutr Diet*. **2020**;77(1):90-102.
210. Yang JM, Luo Y, Zhang JH, Liu QQ, Zhu Q, Ye H, et al. Effects of WB-EMS and protein supplementation on body composition, physical function, metabolism and inflammatory biomarkers in middle-aged and elderly patients with sarcopenic obesity: A meta-analysis of randomized controlled trials. *Exp Gerontol*. **2022**;166:111886.
211. Gielen E, Beckwée D, Delaere A, De Breucker S, Vandewoude M, Bautmans I. Nutritional interventions to improve muscle mass, muscle strength, and physical performance in older people: an umbrella review of systematic reviews and meta-analyses. *Nutr Rev*. **2021**;79(2):121-47.
212. Guo Y, Fu X, Hu Q, Chen L, Zuo H. The Effect of Leucine Supplementation on Sarcopenia-Related Measures in Older Adults: A Systematic Review and Meta-Analysis of 17 Randomized Controlled Trials. *Front Nutr*. **2022**;9:929891.
213. Lee SY, Lee HJ, Lim JY. Effects of leucine-rich protein supplements in older adults with sarcopenia: A systematic review and meta-analysis of randomized controlled trials. *Arch Gerontol Geriatr*. **2022**;102:104758.
214. Liao CD, Wu YT, Tsauo JY, Chen PR, Tu YK, Chen HC, Liou TH. Effects of Protein Supplementation Combined with Exercise Training on Muscle Mass and Function in Older Adults with Lower-Extremity Osteoarthritis: A Systematic Review and Meta-Analysis of Randomized Trials. *Nutrients*. **2020**;12(8).
215. Martínez-Arnau FM, Fonfría-Vivas R, Cauli O. Beneficial Effects of Leucine Supplementation on Criteria for Sarcopenia: A Systematic Review. *Nutrients*. **2019**;11(10).
216. Huang LP, Condello G, Kuo CH. Effects of Milk Protein in Resistance Training-Induced Lean Mass Gains for Older Adults Aged ≥ 60 y: A Systematic Review and Meta-Analysis. *Nutrients*. **2021**;13(8).
217. Kirwan RP, Mazidi M, Rodríguez García C, Lane KE, Jafari A, Butler T, et al. Protein interventions augment the effect of resistance exercise on appendicular lean mass and handgrip strength in older adults: a systematic review and meta-analysis of randomized controlled trials. *Am J Clin Nutr*. **2022**;115(3):897-913.
218. Liao CD, Chen HC, Huang SW, Liou TH. The Role of Muscle Mass Gain Following Protein Supplementation Plus Exercise Therapy in Older Adults with Sarcopenia and Frailty Risks: A Systematic Review and Meta-Regression Analysis of Randomized Trials. *Nutrients*. **2019**;11(8).
219. Nunes EA, Colenso-Semple L, McKellar SR, Yau T, Ali MU, Fitzpatrick-Lewis D, et al. Systematic review and meta-analysis of protein intake to support muscle mass and function in healthy adults. *J Cachexia Sarcopenia Muscle*. **2022**;13(2):795-810.
220. Coelho-Junior HJ, Calvani R, Picca A, Tosato M, Landi F, Marzetti E. Protein Intake and Frailty in Older Adults: A Systematic Review and Meta-Analysis of Observational Studies. *Nutrients*. **2022**;14(13).
221. Yang J, Zhang Y, Na X, Zhao A. β-Carotene Supplementation and Risk of Cardiovascular Disease: A Systematic Review and Meta-Analysis of Randomized Controlled Trials. *Nutrients*. **2022**;14(6).
222. Chen F, Wang J, Cheng Y, Li R, Wang Y, Chen Y, et al. Magnesium and Cognitive Health in Adults: A Systematic Review and Meta-Analysis. *Adv Nutr*. **2024**;15(8):100272.
